# Author Correction: Divalent anion-driven framework regulation in Zr-based halide solid electrolytes for all-solid-state batteries

**DOI:** 10.1038/s41467-026-68882-7

**Published:** 2026-01-29

**Authors:** Jae-Seung Kim, Daseul Han, Jinyeong Choe, Youngkyung Kim, Hae-Yong Kim, Soeul Lee, Jiwon Seo, Seung-Hui Ham, You-Yeob Song, Chang-Dae Lee, Juho Lee, Hiram Kwak, Jinsoo Kim, Yoon Seok Jung, Sung-Kyun Jung, Kyung-Wan Nam, Dong-Hwa Seo

**Affiliations:** 1 Department of Materials Science and Engineering, Korea Advanced Institute of Science and Technology (KAIST), Daejeon, Republic of Korea; 2 Department of Energy and Materials Engineering, Dongguk University, Seoul, Republic of Korea; 3 Department of Materials Science and Engineering, Seoul National University (SNU), Seoul, Republic of Korea; 4 Department of Energy Science and Engineering, Daegu Gyeongbuk Institute of Science and Technology (DGIST), Daegu, Republic of Korea; 5 Department of Chemical and Biomolecular Engineering, Yonsei University, Seoul, Republic of Korea; 6 School of Transdisciplinary Innovations, Seoul National University (SNU), Seoul, Republic of Korea; 7 Research Institute of Advanced Materials (RIAM), Seoul National University (SNU), Seoul, Republic of Korea; 8 Institute for Rechargeable Battery Innovations Research, Seoul National University (SNU), Seoul, Republic of Korea

**Keywords:** Batteries, Batteries

Correction to: *Nature Communications* 10.1038/s41467-025-65702-2, published online 27 November 2025

In the version of the article initially published, Yoon Seok Jung’s name was incorrectly hyphenated (appearing as Yoon-Seok) and has now been corrected in the HTML and PDF versions of the article.

