# Supplementary material for: Divalent anion-driven framework regulation in Zr-based halide solid electrolytes for all-solid-state batteries
Source: Nat Commun. 2025 Nov 27;16:10678. doi: 10.1038/s41467-025-65702-2 (PMC12660725; doi:10.1038/s41467-025-65702-2)
Supplement: Supplementary file 1 — Supplementary Information [file 41467_2025_65702_MOESM1_ESM.pdf]

[Supplementary Information]

Divalent Anion-Driven Framework Regulation in Zr-Based Halide Solid Electrolytes for All-Solid-State Batteries

Jae-Seung Kim,[a†] Daseul Han,[b†] Jinyeong Choe,[a] Youngkyung Kim,[c] Hae-Yong Kim,[b] Soeul Lee,[b] Jiwon Seo,[a] Seung-Hui Ham,[a] You-Yeob Song,[a] Chang-Dae Lee,[a] Juho Lee,[d] Hiram Kwak,[e] Jinsoo Kim,[d] Yoon-Seok Jung,[e] Sung-Kyun Jung,*[c] Kyung-Wan Nam,*[b] and Dong-Hwa Seo*[a]*

[a] *Department of Materials Science and Engineering, Korea Advanced Institute of Science and Technology (KAIST), Daejeon, 34141, Republic of Korea*

[b] *Department of Energy and Materials Engineering, Dongguk University, Seoul, 04620, Republic of Korea*

[c] *Department of Materials Science and Engineering, Seoul National University (SNU), Seoul, 08826, Republic of Korea*

[d] *Department of Energy Science and Engineering, Daegu Gyeongbuk Institute of Science and Technology (DGIST), Daegu, 42988, Republic of Korea*

[e] *Department of Chemical and Biomolecular Engineering, Yonsei University, Seoul, 03722, Republic of Korea*

† *These authors contributed equally: Jae-Seung Kim, Daseul Han.*

Corresponding Authors: *e-mail: yoonsjung@yonsei.ac.kr (Prof. Y. S. Jung), naecard@snu.ac.kr (Prof. S.-K. Jung), knam@dongguk.edu (Prof. K.-W. Nam), dseo@kaist.ac.kr (Prof. D.-H. Seo)

Table of Contents

1. Supplementary Figures
2. Supplementary Tables and Supplementary Text

1. Supplementary Figures

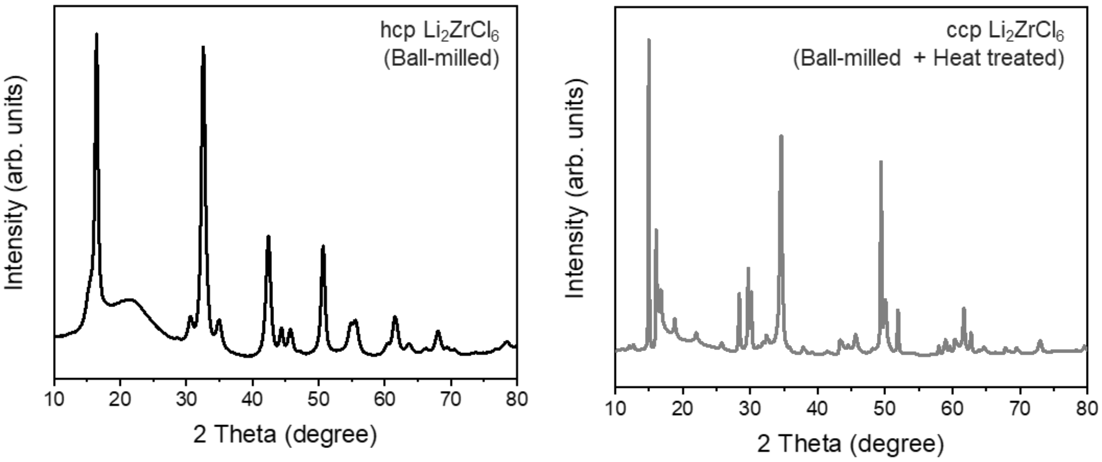

Supplementary Fig. 1. Synchrotron XRD patterns of hexagonal close-packed (hcp)-Li_2ZrCl_6 (left) and cubic close-packed (ccp)-Li_2ZrCl_6 (right).

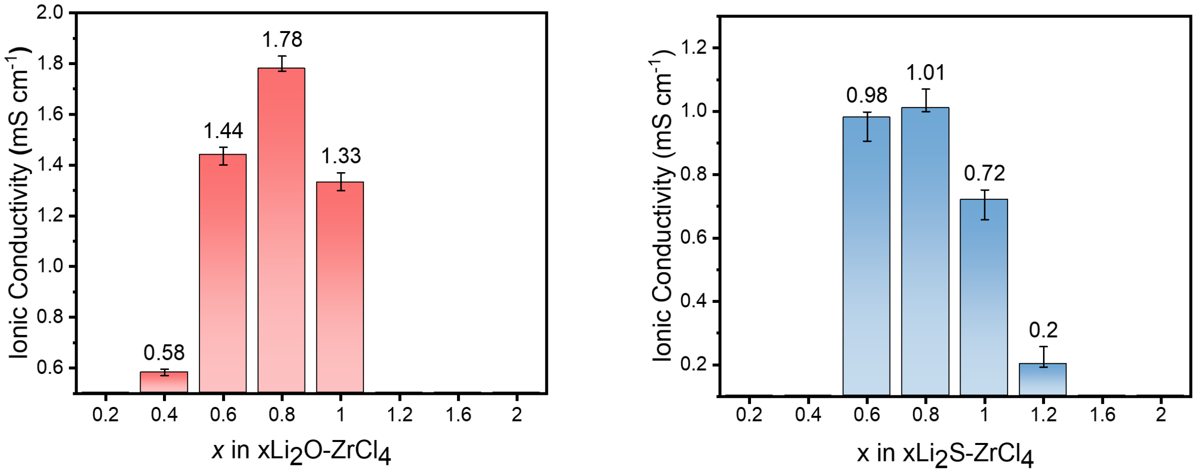

Supplementary Fig. 2. Ionic conductivities of $x$$Li_2O$–$ZrCl_4$ and $x$$Li_2S$–$ZrCl_4$. Error bars represent the standard deviation from n = 4 independent ionic conductivity measurements.

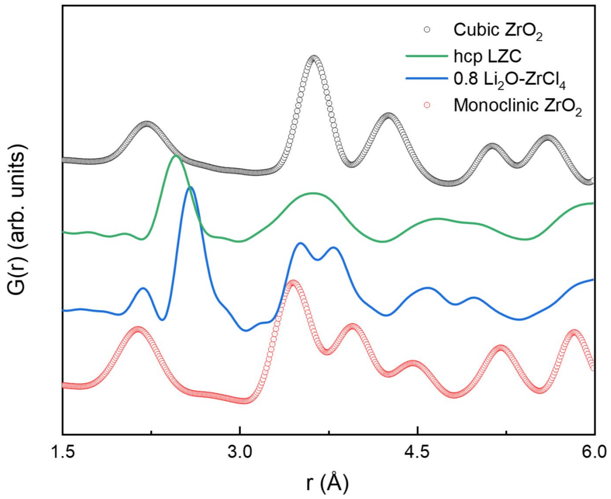

Supplementary Fig. 3. Observed and calculated PDF fitting curves for $0.8Li_2O–ZrCl_4$ compared with cubic ZrO_2, monoclinic ZrO_2 and hcp-Li_2ZrCl_6 phase. The detailed results are summarized in **Supplementary Text 1**.

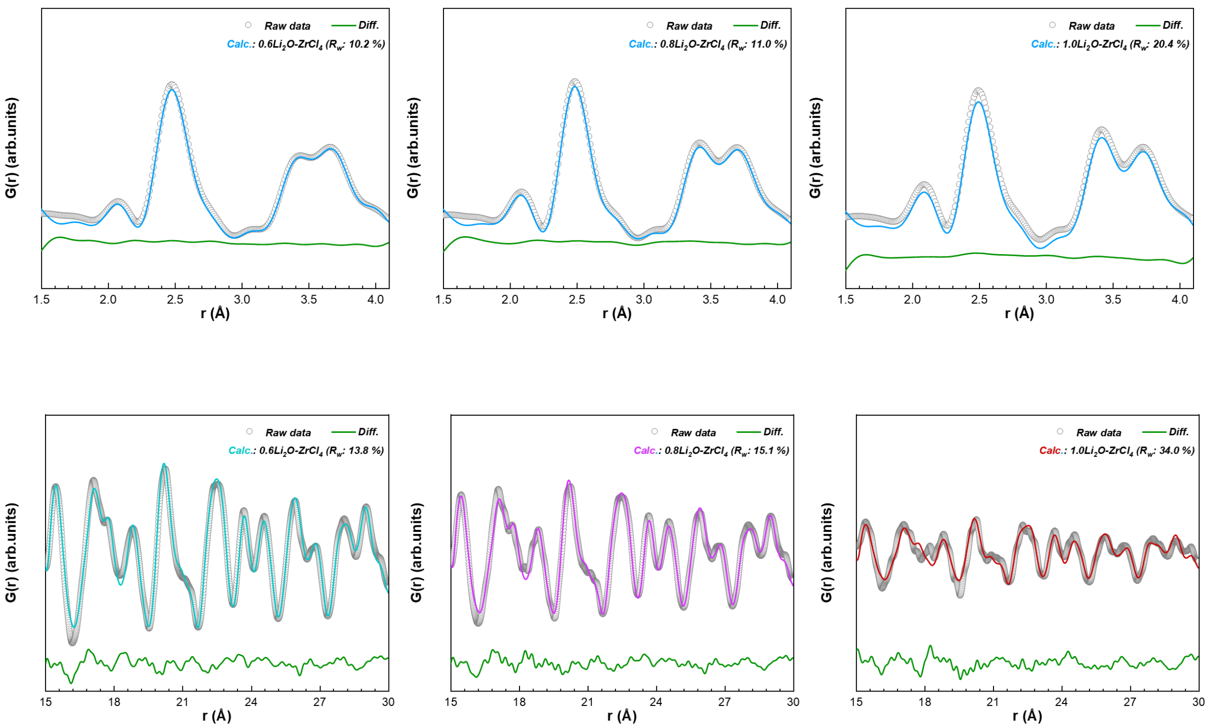

Supplementary Fig. 4. Observed and calculated PDF short-range region (1.5-4.1 Å, top) and long-range region (15-30 Å, bottom) fitting curves for 0.6Li_2O–$ZrCl_4$, 0.8Li_2O–$ZrCl_4$ and 1.0Li_2O–$ZrCl_4$. The detailed results are summarized in **Supplementary Table 1-6**.

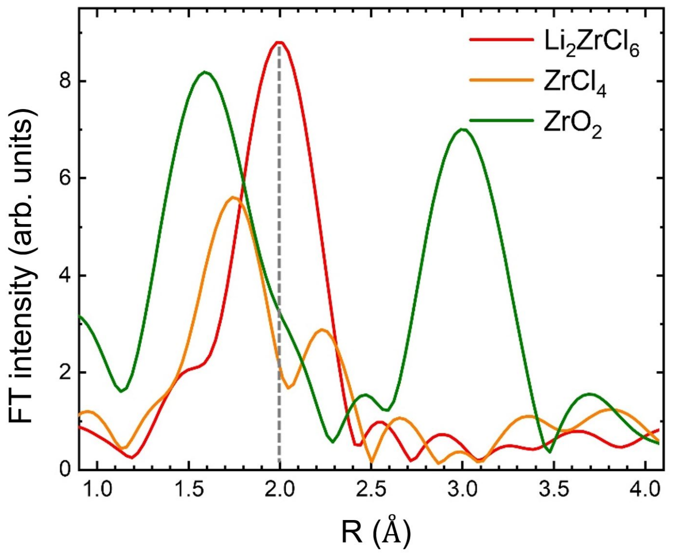

Supplementary Fig. 5. Zr K-edge EXAFS fitting data of hcp-Li_2ZrCl_6, $ZrCl_4$, and ZrO_2.

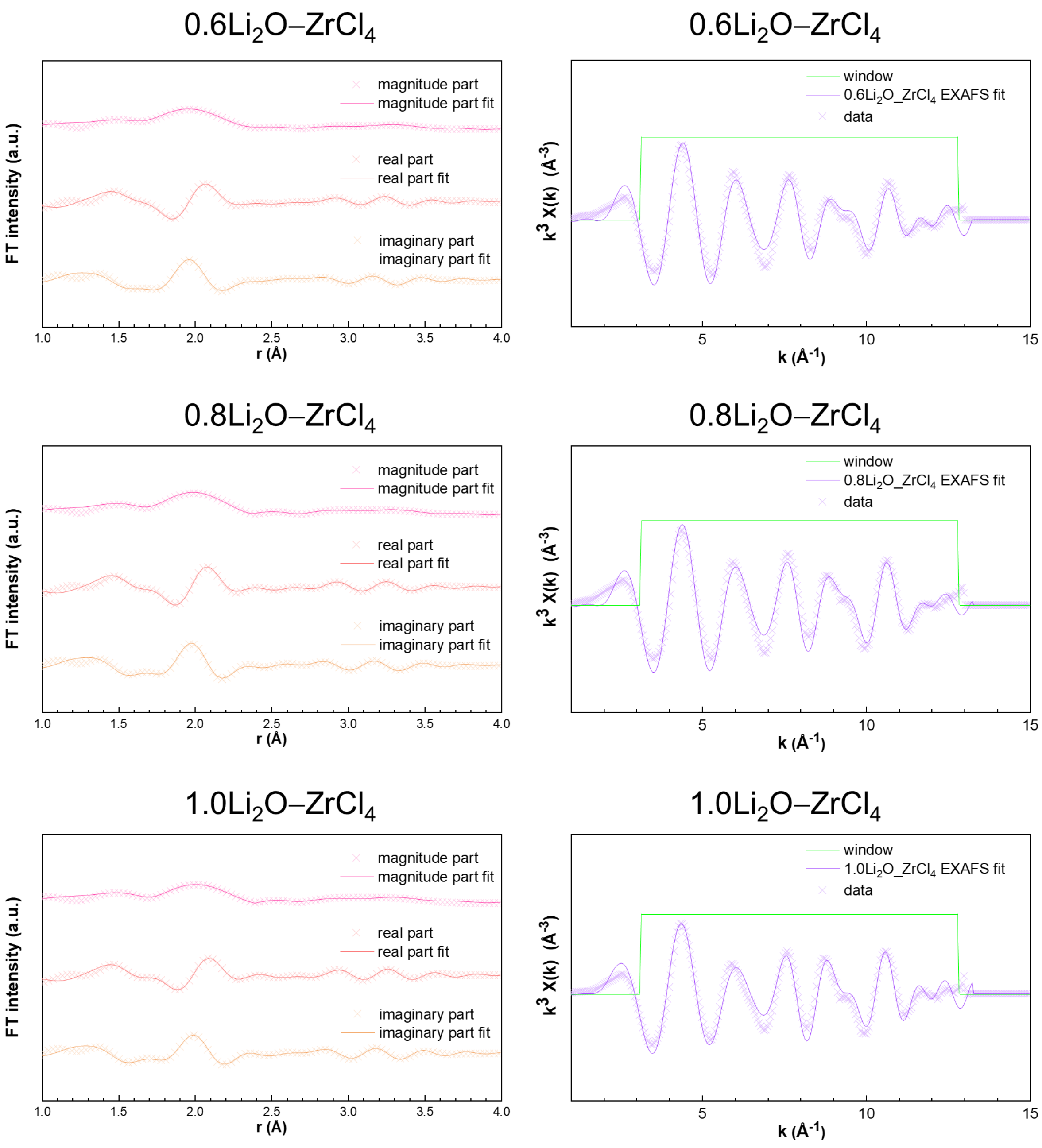

Supplementary Fig. 6. Zr K-edge EXAFS result**s.** The real/imaginary part of FT (left) and $k^3\chi(k)$ (right) based on R-space curve fitting of xLi_2O–$ZrCl_4$ (x = 0.6, 0.8, 1.0). The detailed results are summarized in **Supplementary Table 7**.

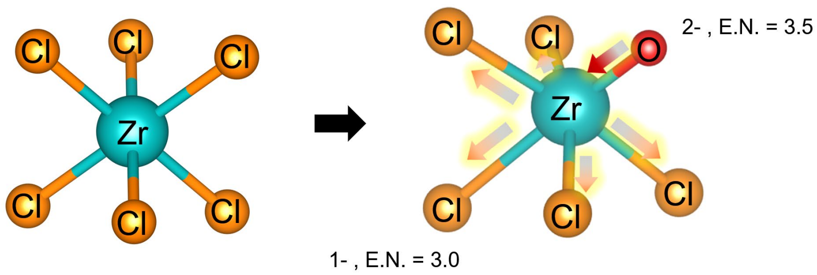

Supplementary Fig. 7. Bonding features of oxygen-substituted Zr octahedron. Electronegativity (E.N.) and negative charge affected electron charge density in central Zr metal.

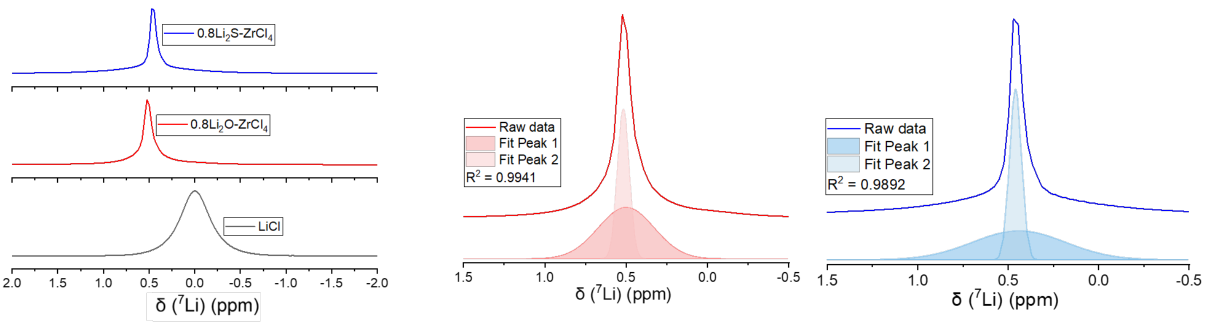

Supplementary Fig. 8. ^{7}Li-MAS NMR spectra of $0.8Li_2A–ZrCl_4$ (A=O, S) and LiCl.

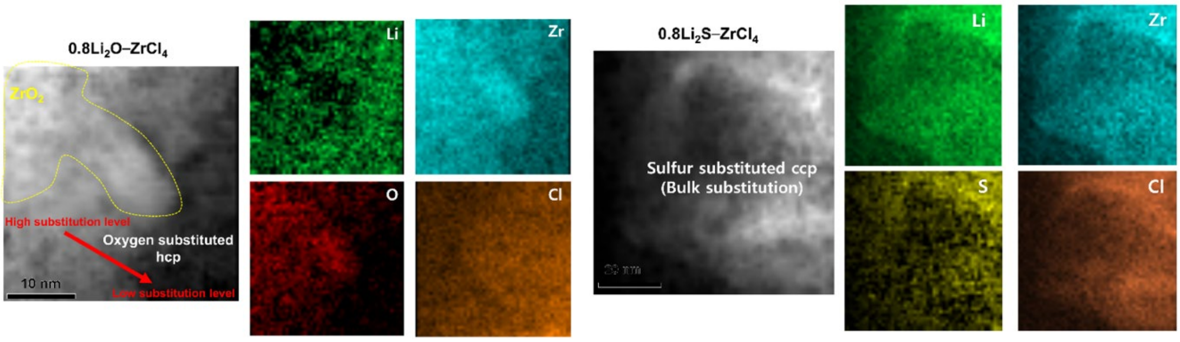

Supplementary Fig. 9. Scanning transmission electron microscopy (STEM) image and electron energy loss spectroscopy (EELS) elemental mapping (Li, Zr, Cl, O, S) of $0.8Li_2O$–$ZrCl_4$ (left) and $0.8Li_2S$–$ZrCl_4$ (right).

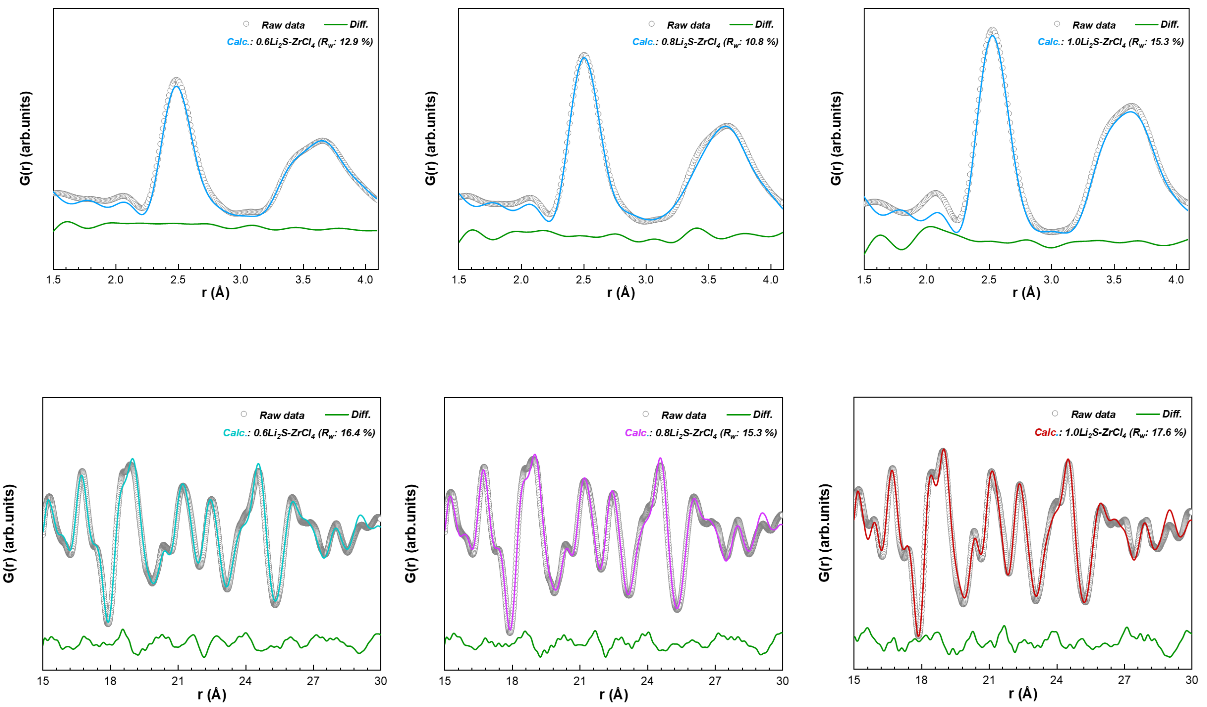

Supplementary Fig. 10. Observed and calculated PDF short-range region (1.5-4.1 Å, top) and long-range region (15-30 Å, bottom) fitting curves for $0.6Li_2S–ZrCl_4$, $0.8Li_2S–ZrCl_4$ and $1.0Li_2S–ZrCl_4$. The detailed results are summarized in **Supplementary Table 8-13**.

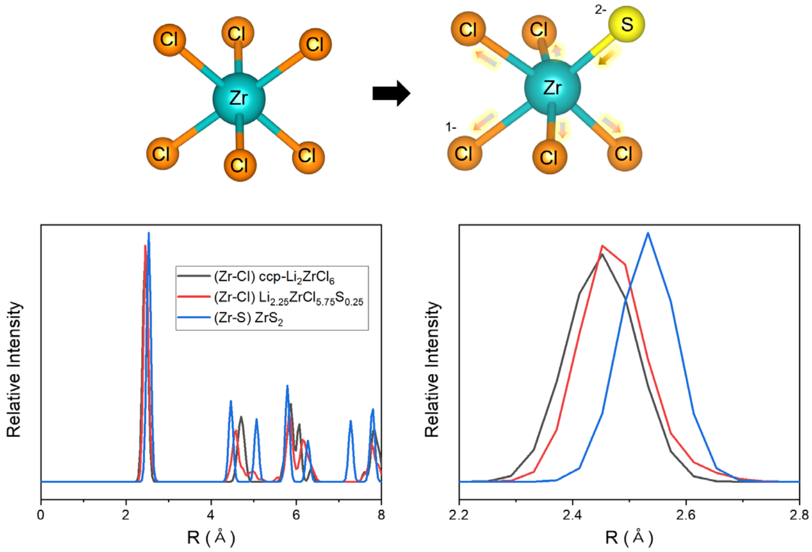

Supplementary Fig. 11. Bonding features of sulfur-substituted Zr octahedron and simulated radial distribution function (RDF) of ccp-Li_2ZrCl_6, sulfur-substituted ccp-$Li_{2.25}ZrCl_{5.75}S_{0.25}$, and ZrS_2. The structures were obtained by DFT calculations, and the lattices are fully relaxed with the same input parameters.

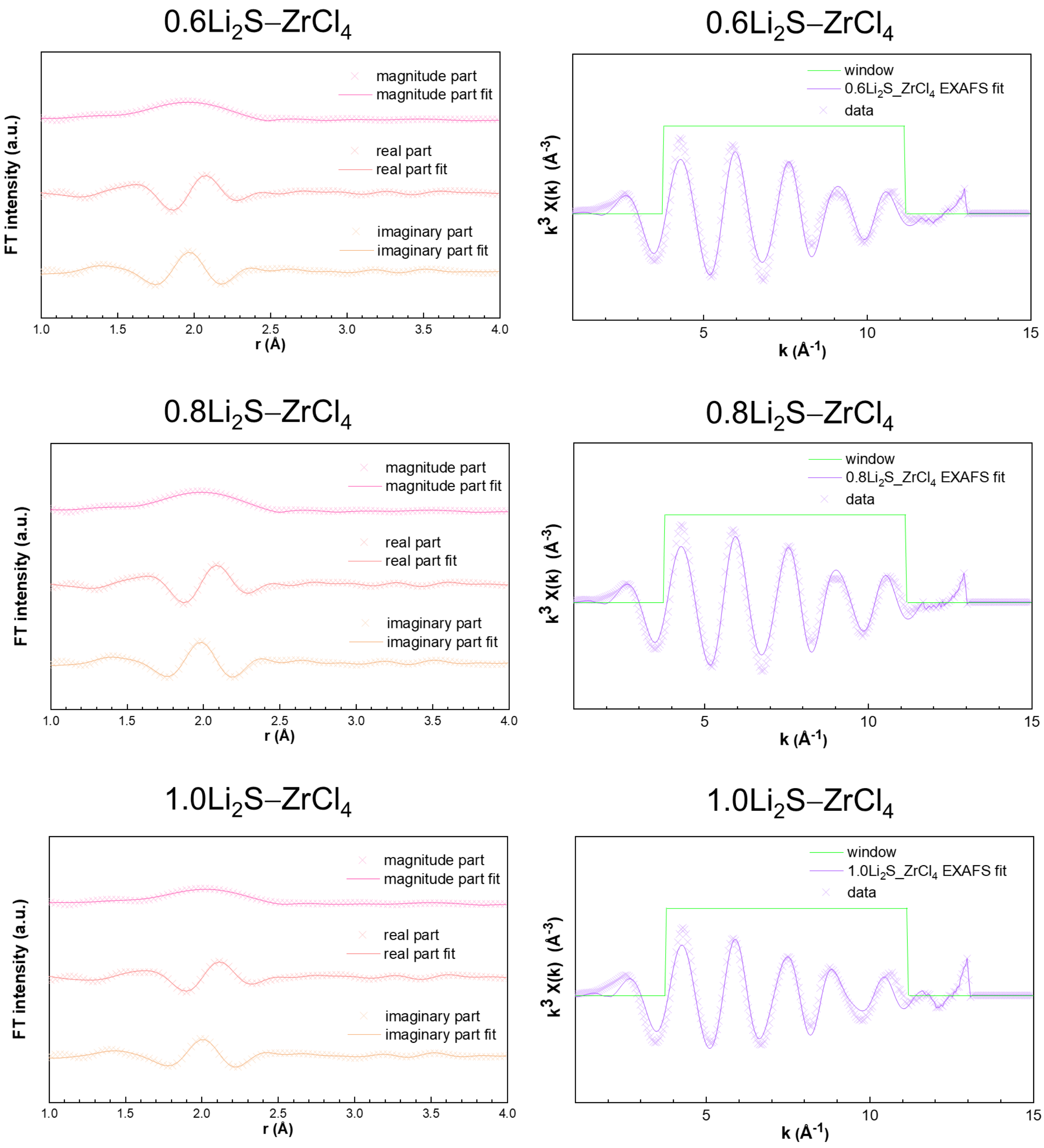

Supplementary Fig. 12. Zr K-edge EXAFS results. The real/imaginary part of FT (left) and $k^3\chi(k)$ (right) based on R-space curve fitting of xLi$_2$S–ZrCl$_4$ (x = 0.6, 0.8, 1.0). The detailed results are summarized in **Supplementary Table 14**.

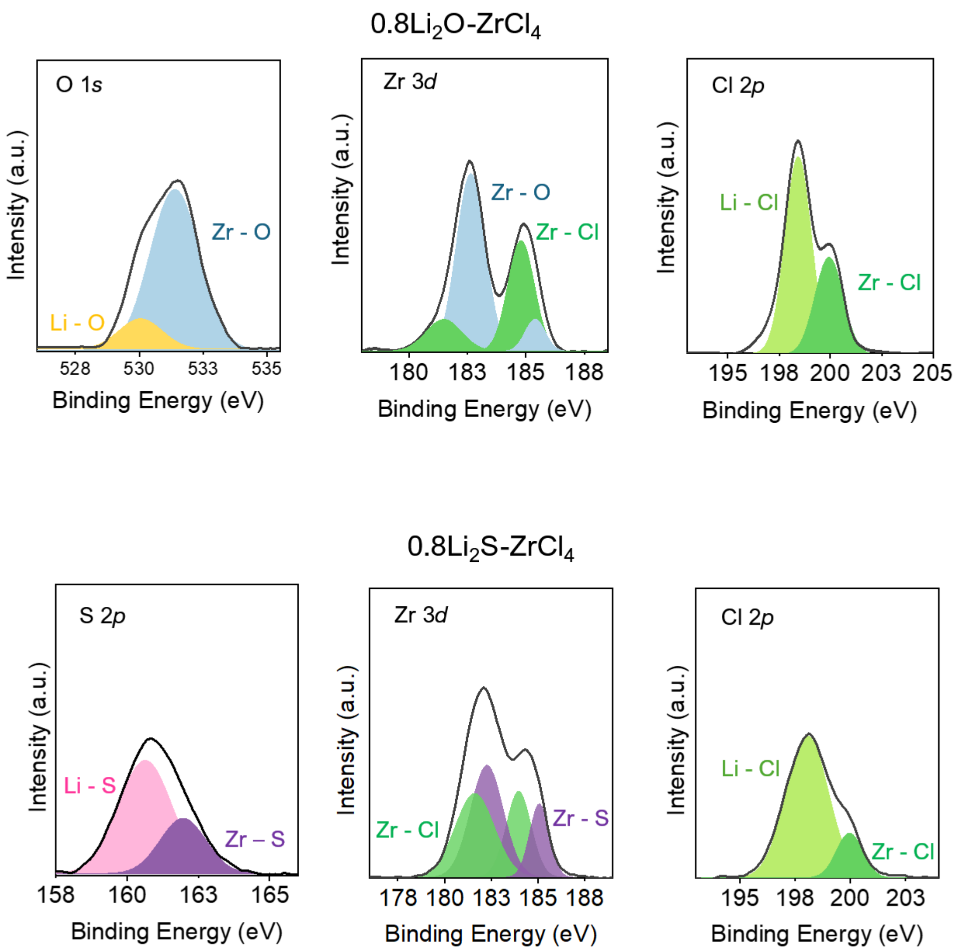

Supplementary Fig. 13. XPS spectra of $0.8Li_2O$–$ZrCl_4$ (O 1*s*, Zr 3*d*, Cl 2*p*) and $0.8Li_2S$–$ZrCl_4$ (S 2*p*, Zr 3*d*, Cl 2*p*).

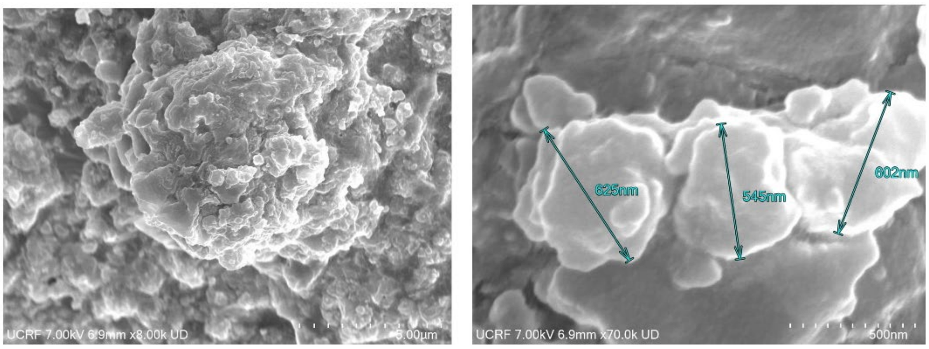

Supplementary Fig. 14. SEM images of $0.8Li_2O–ZrCl_4$.

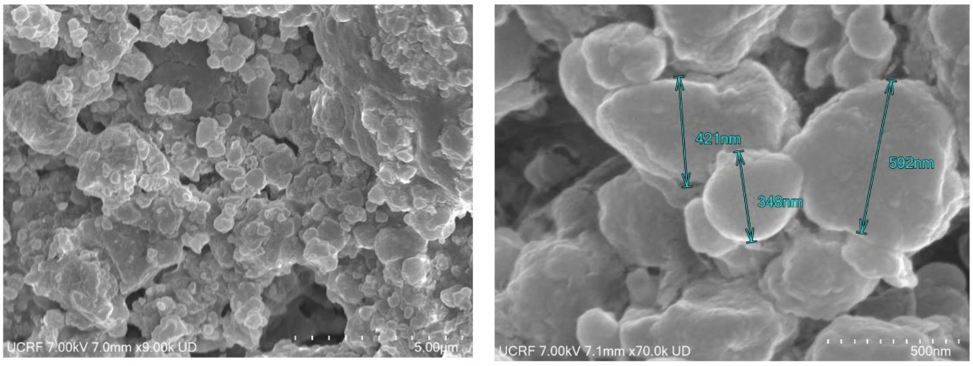

Supplementary Fig. 15. SEM images of $0.8Li_2S–ZrCl_4$.

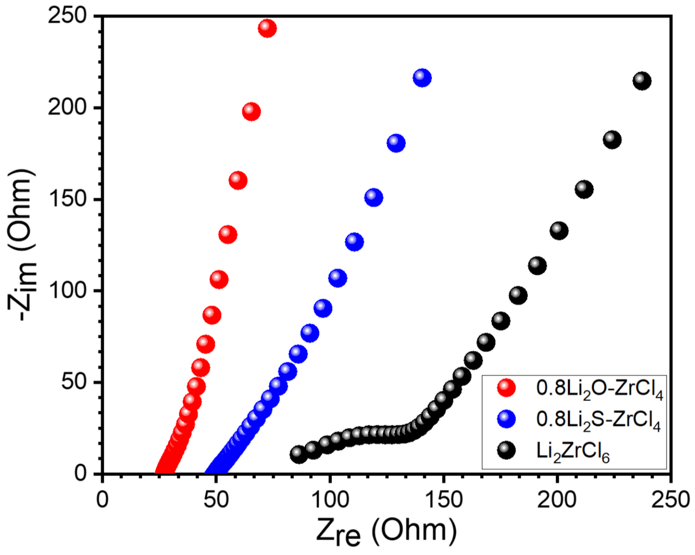

Supplementary Fig. 16. Nyquist plots of EIS results for $0.8Li_2O$–$ZrCl_4$, $0.8Li_2S$–$ZrCl_4$, and hcp-Li_2ZrCl_6.

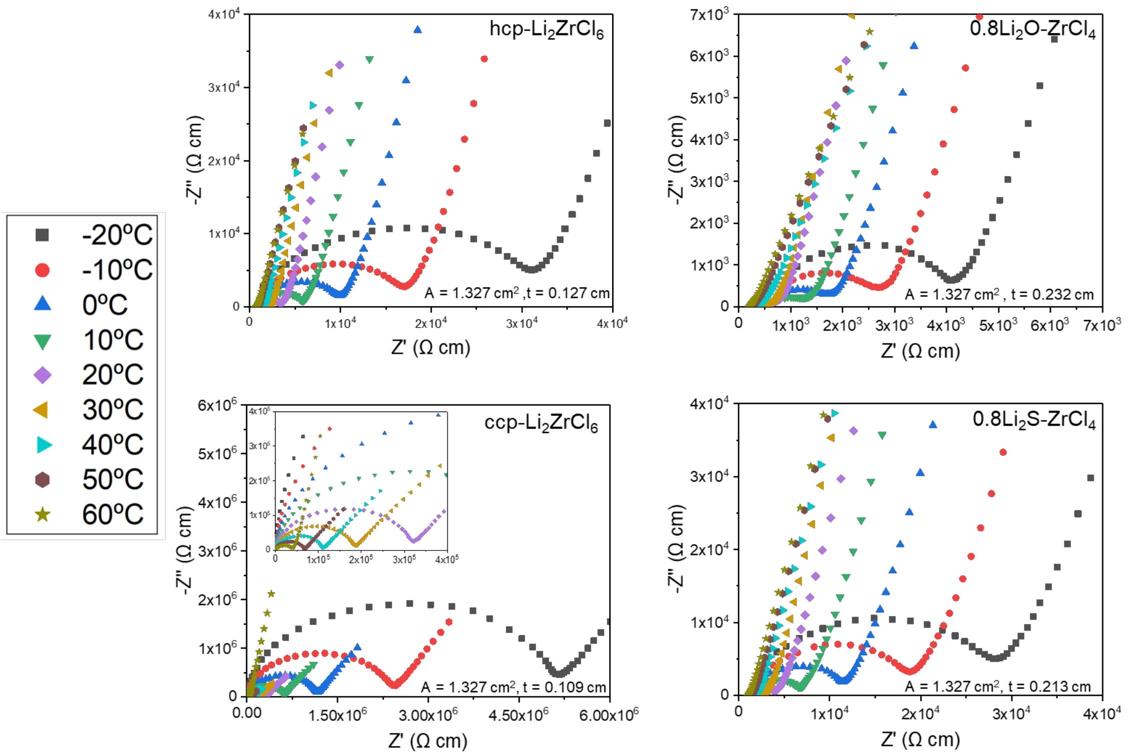

Supplementary Fig. 17. Nyquist plots of EIS results for hcp-Li_2ZrCl_6, 0.8Li_2O–$ZrCl_4$, ccp-Li_2ZrCl_6 and 0.8Li_2S–$ZrCl_4$ measured at various temperatures ranging from –20 °C to 60 °C.

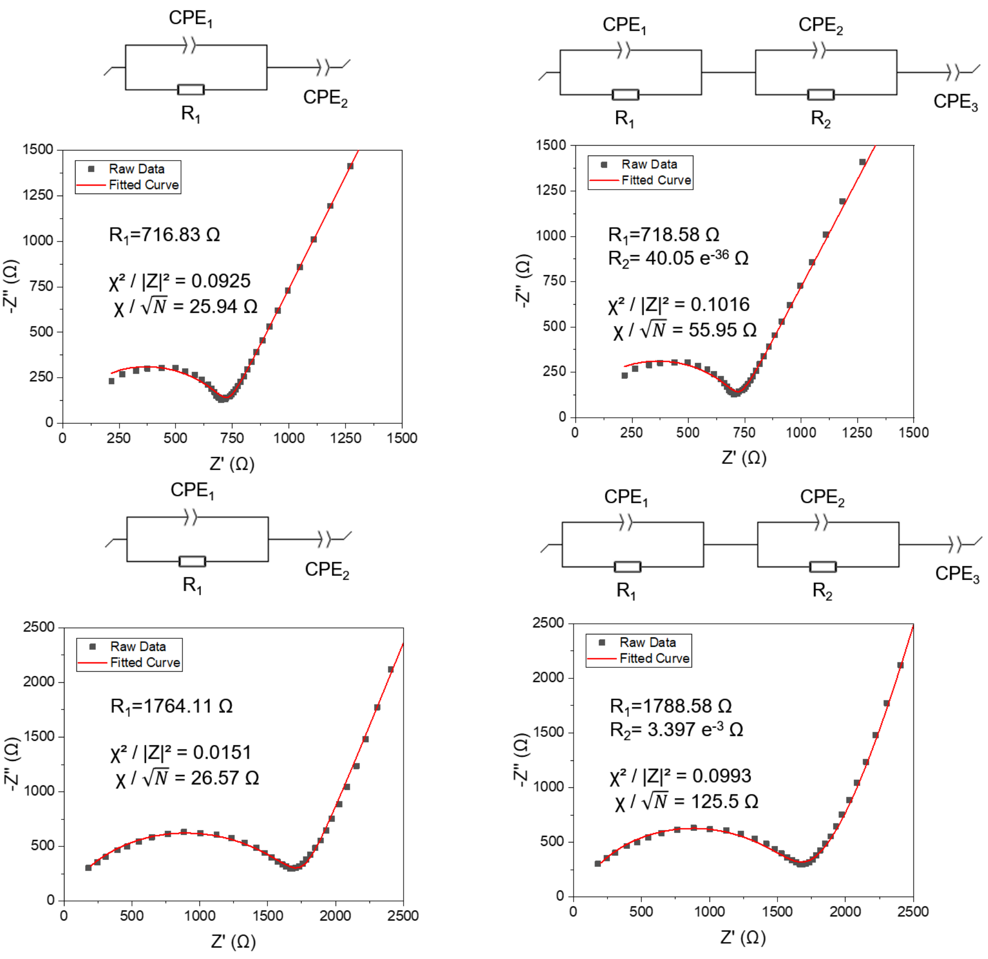

Supplementary Fig. 18. Comparison of Nyquist plot with equivalent circuit fitting using single-phase and multi-component models for 0.8Li_2O–$ZrCl_4$ (top) and 0.8Li_2S–$ZrCl_4$ (bottom) at -20°C. Equivalent circuit fitting confirms that no significant differences were observed among the fitting models, and the fitting parameters are not intended for quantitative discussion.

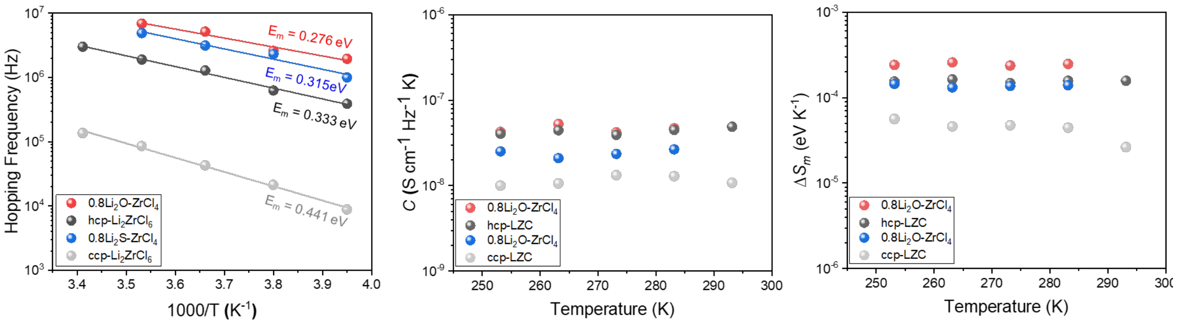

Supplementary Fig. 19. Arrhenius plots of hopping frequency, carrier concentration factor, migration entropy of hcp-Li_2ZrCl_6, 0.8Li_2O–$ZrCl_4$, ccp-Li_2ZrCl_6 and 0.8Li_2S–$ZrCl_4$.

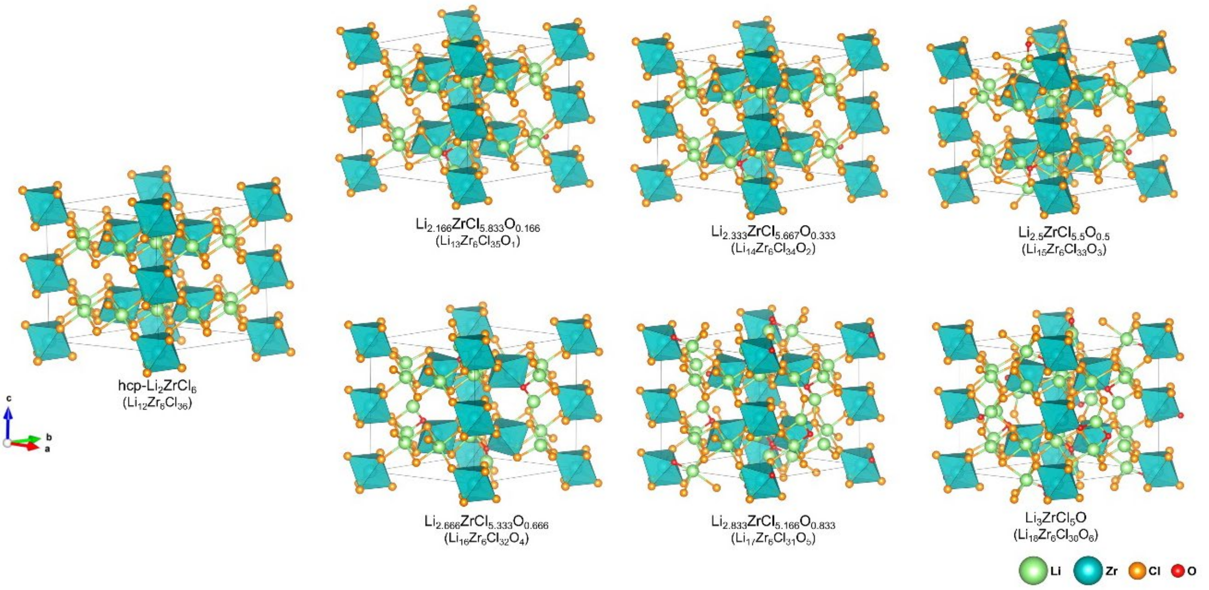

Supplementary Fig. 20. Crystal structures of hcp-$Li_{2+x}ZrCl_{6-x}O_x$ (x = 0, 0.166, 0.333, 0.5, 0.667, 0.833, 1). All the structures have been fully relaxed using the same DFT parameters.

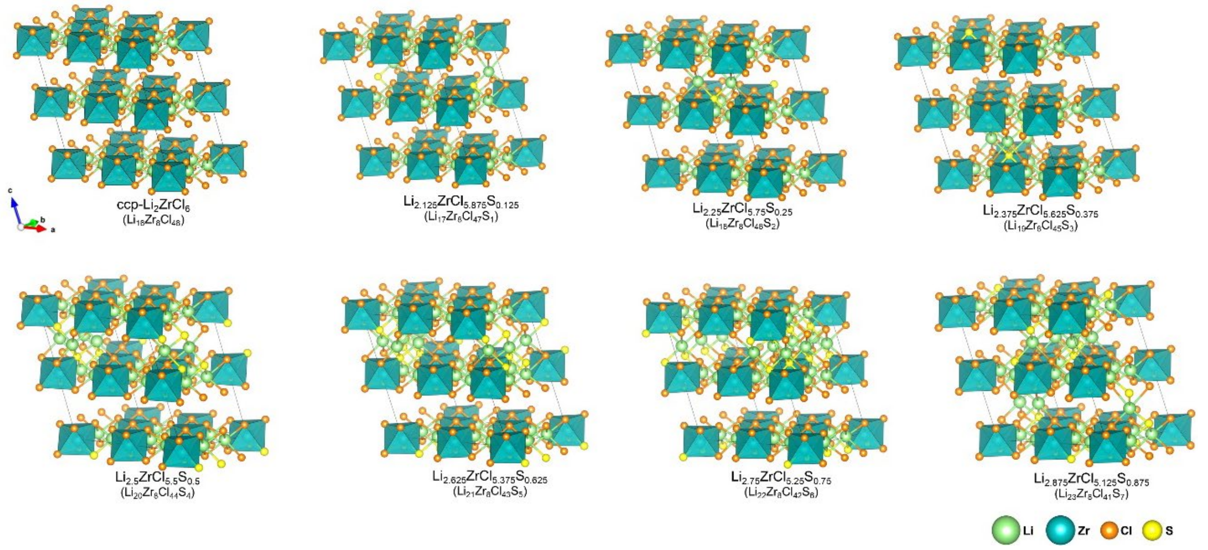

Supplementary Fig. 21. Crystal structures of ccp-$Li_{2+x}ZrCl_{6-x}S_x$ (x = 0, 0.125, 0.25, 0.375, 0.5, 0.625, 0.75, 0.875, 1). All the structures have been fully relaxed using the same DFT parameters.

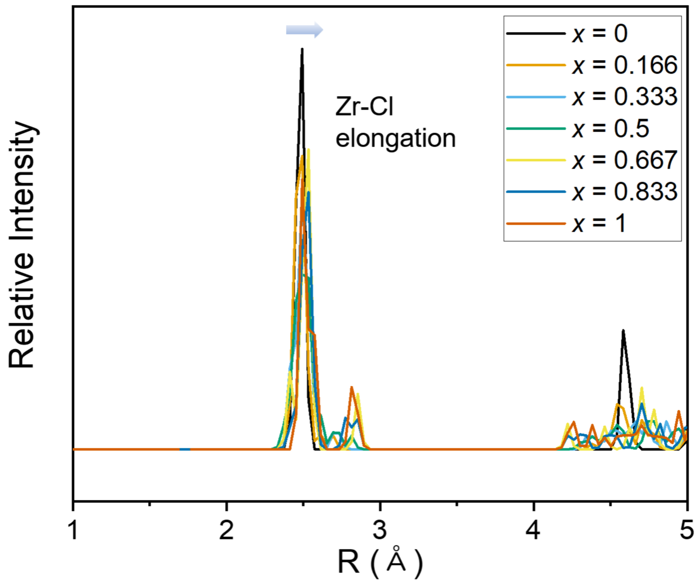

Supplementary Fig. 22. Simulated RDF of $Li_{2+x}ZrCl_{6-x}O_x$ (x = 0, 0.166, 0.333, 0.5, 0.667, 0.833, and 1).

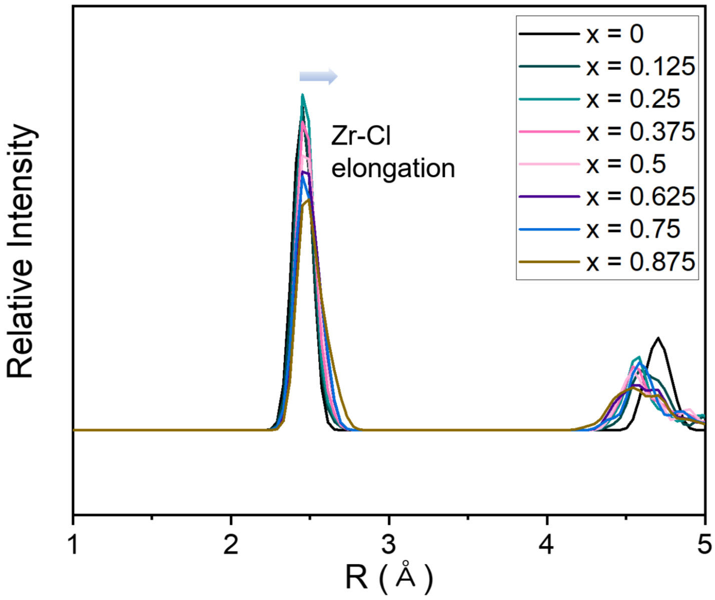

Supplementary Fig. 23. Simulated RDF of ccp-$Li_{2+x}ZrCl_{6-x}S_x$ (x = 0, 0.125, 0.25, 0.375, 0.5, 0.625, 0.75, 0.875).

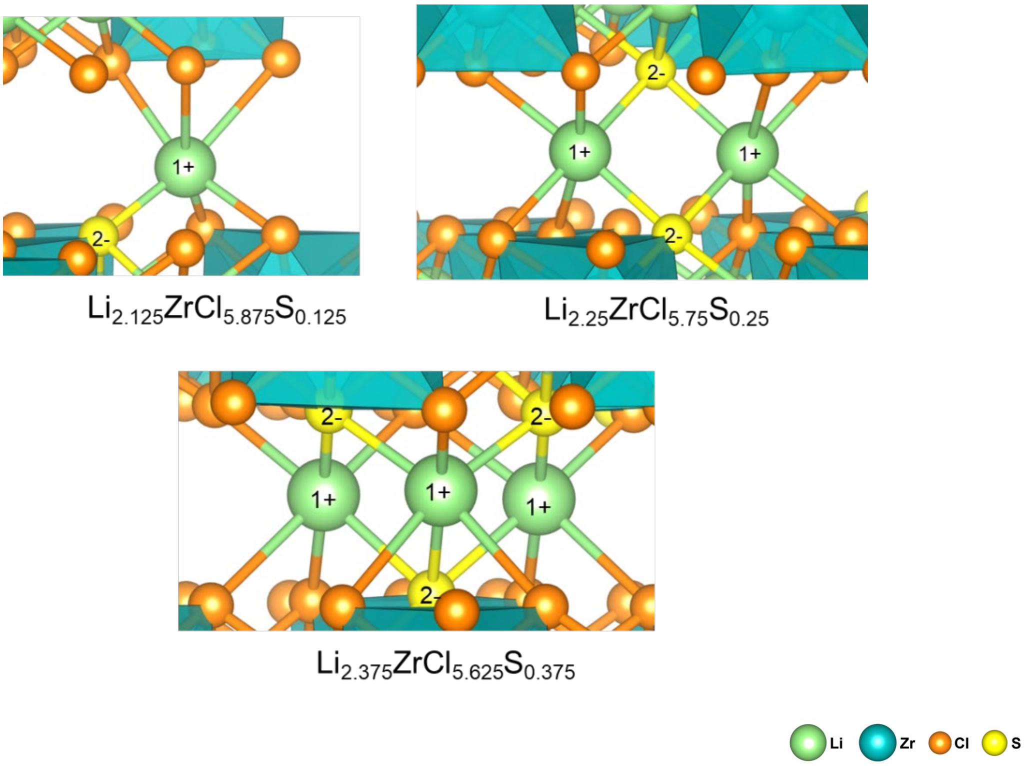

Supplementary Fig. 24. Coordination structure of additional Li in the most stable structure of $Li_{2.125}ZrCl_{5.875}S_{0.125}$, $Li_{2.25}ZrCl_{5.75}S_{0.25}$, and $Li_{2.375}ZrCl_{5.625}S_{0.375}$.

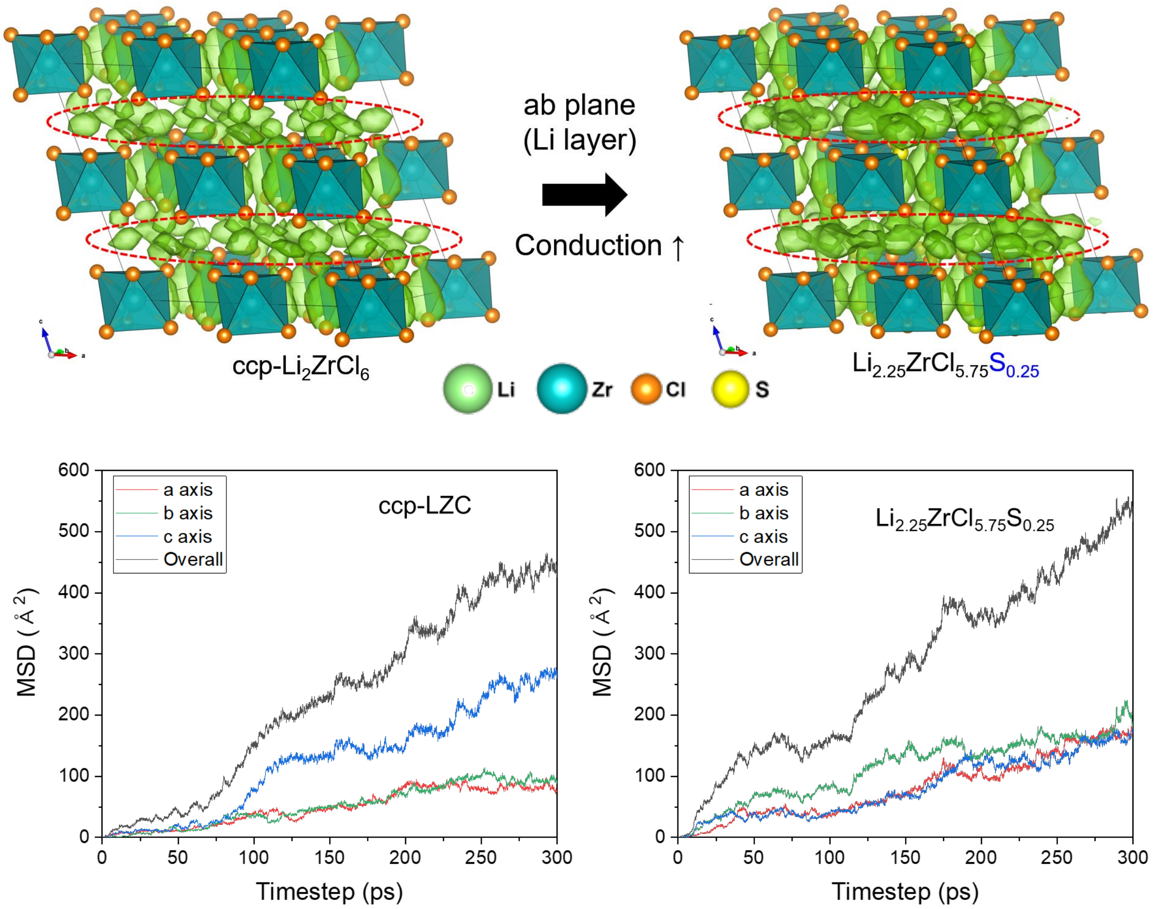

Supplementary Fig. 25. Li probability density at 600 K in ~300 ps (isosurface value P = P_{max}/100) of ccp-Li_2ZrCl_6 (top-left) and ccp-$Li_{2.25}ZrCl_{5.75}S_{0.25}$ (top-right). Mean square displacement (MSD) of AIMD simulations at 600 K in ~300 ps of ccp-Li_2ZrCl_6 (down-left) and ccp-$Li_{2.25}ZrCl_{5.75}S_{0.25}$ (down-right). Sulfur incorporation induces lattice expansion and structural distortion in the ccp framework, activating three-dimensional ionic conduction otherwise limited to the ab plane.

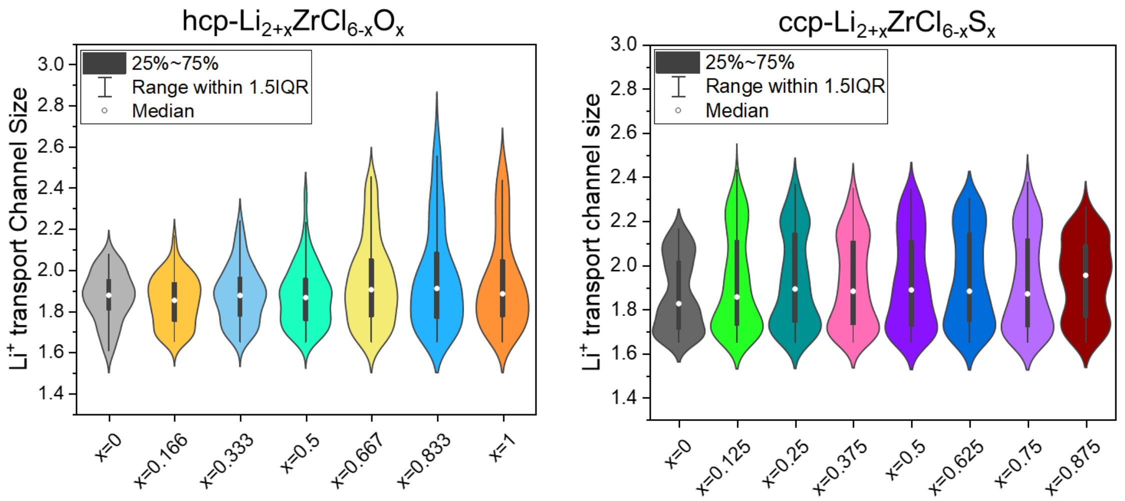

Supplementary Fig. 26. Topological analysis and Li^+-transport channel size of hcp-$Li_{2+x}ZrCl_{6-x}O_x$ (x=0, 0.166, 0.333, 0.5, 0.667, 0.833, and 1) and ccp-$Li_{2+x}ZrCl_{6-x}S_x$ (x = 0, 0.125, 0.25, 0.375, 0.5, 0.625, 0.75, 0.875). White dots indicate the median, boxes represent the interquartile range (25–75%), and whiskers extend to 1.5× the interquartile range.

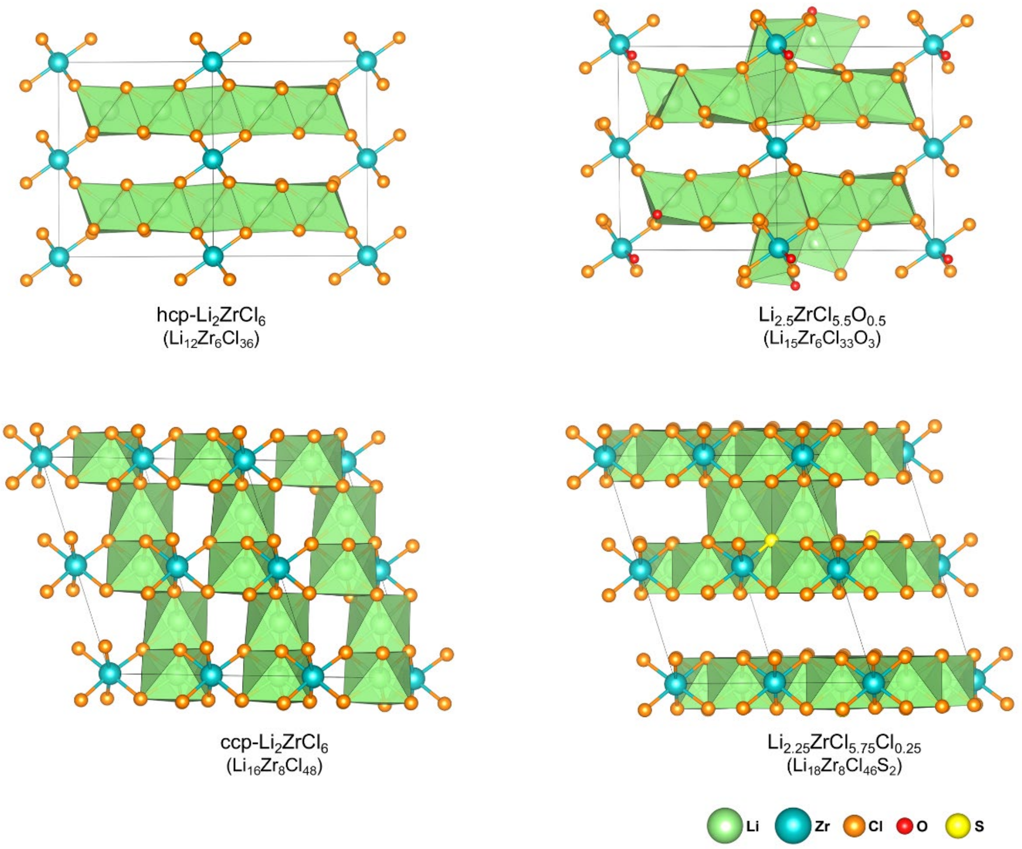

Supplementary Fig. 27. Li polyhedral features in crystal structure of hcp-Li_2ZrCl_6 with $Li_{2.5}ZrCl_{5.5}O_{0.5}$ and ccp-Li_2ZrCl_6 with $Li_{2.25}ZrCl_{5.75}S_{0.25}$.

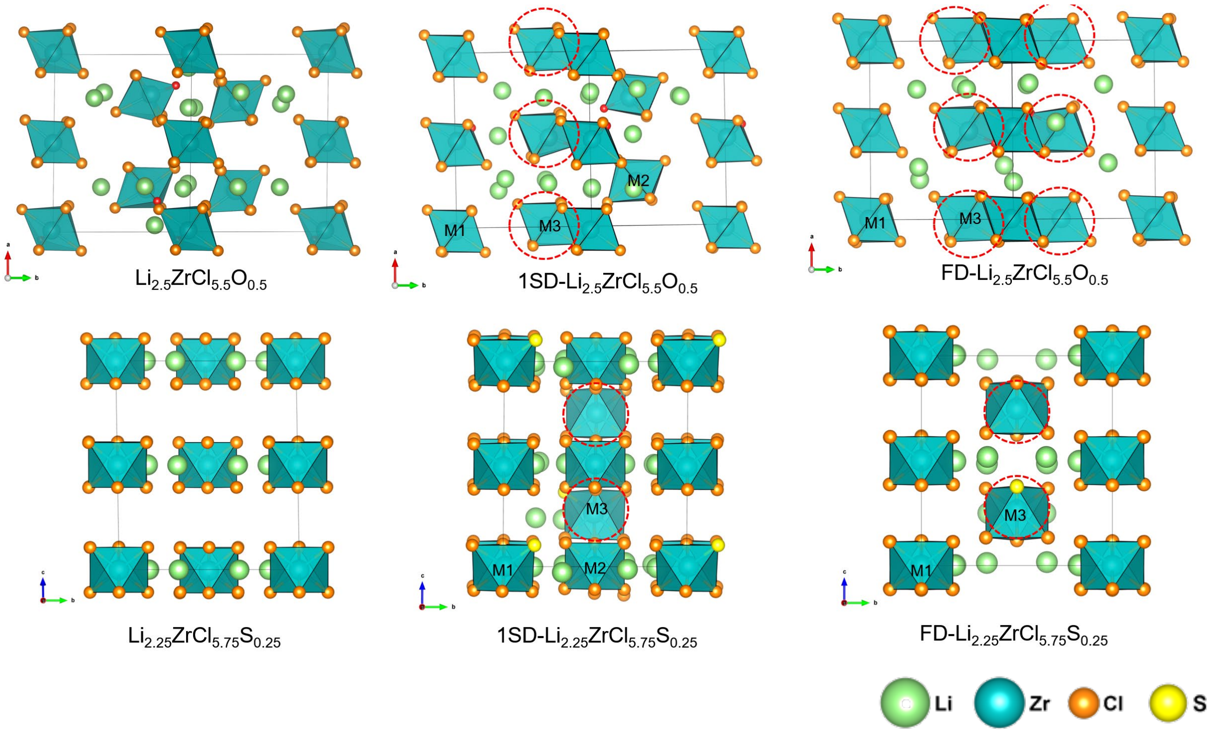

Supplementary Fig. 28. Crystal structures depending on metal ordering in hcp structure ($Li_{2.5}ZrCl_{5.5}O_{0.5}$) and ccp structure ($Li_{2.25}ZrCl_{5.75}S_{0.25}$). The red circle shows a disordered metal site in lattice structure. 1 side-disorder (1SD) means cation disorder occurs asymmetrically near one side of the M2/M3 sites, and M3-site fully disordered (FD) indicates that the M3 sites are entirely filled with metal cations.

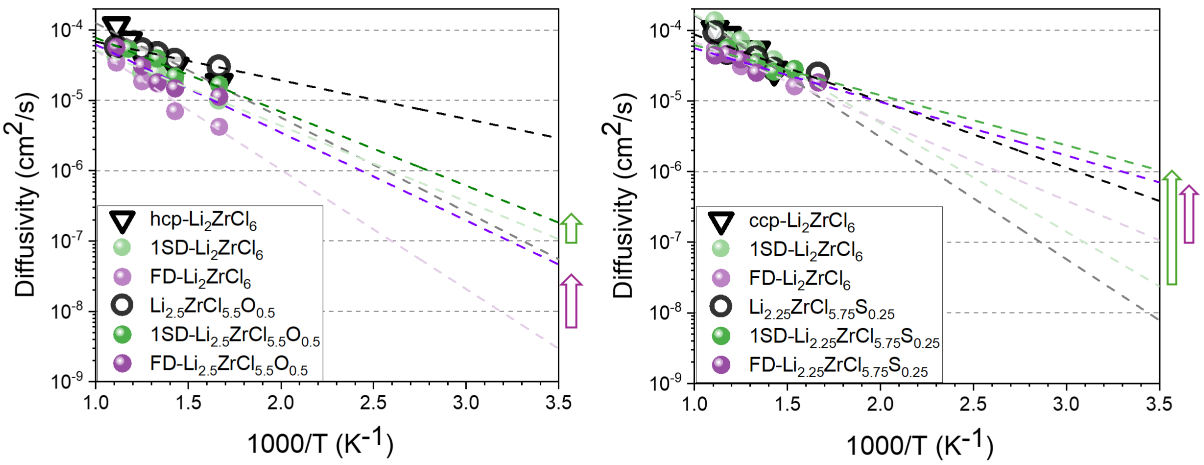

Supplementary Fig. 29. Arrhenius plots obtained from ab initio molecular dynamics (AIMD) simulations of ordered-hcp (black), 1SD-hcp (green) and FD-hcp (purple) structures in Li_2ZrCl_6 and $Li_{2.5}ZrCl_{5.5}O_{0.5}$ compositions (left). Arrhenius plots of the AIMD simulation of ordered-ccp (black), 1SD-ccp (green) and FD-ccp (purple) in Li_2ZrCl_6 and $Li_{2.25}ZrCl_{5.75}S_{0.25}$ compositions (right). 1 side-disorder (1SD) means cation disorder occurs asymmetrically near one side of the M2/M3 sites, and M3-site fully disordered (FD) indicates that the M3 sites are entirely filled with metal cations.

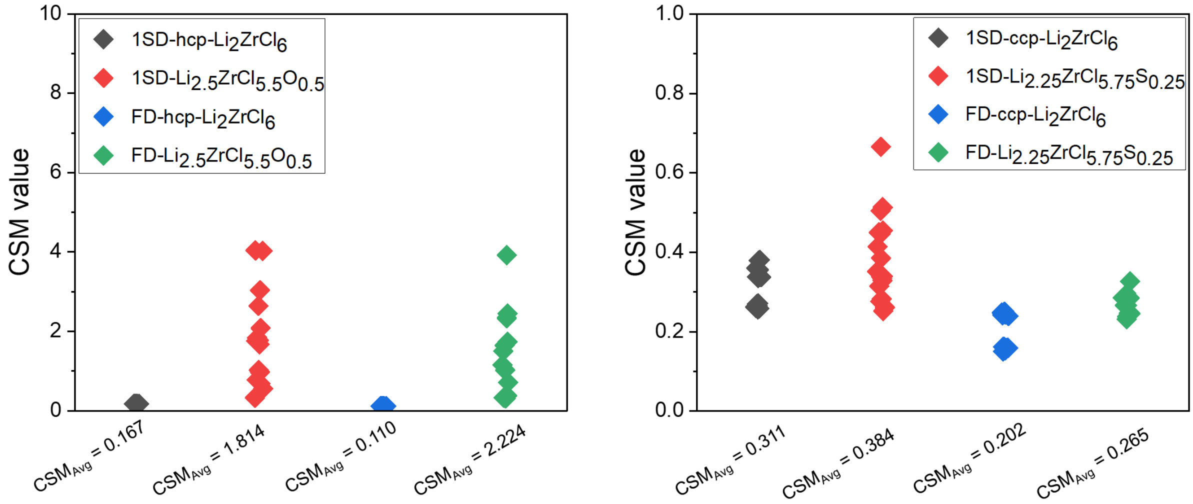

Supplementary Fig. 30. Distribution of continuous symmetry measured (CSM) value of Li octahedral site in 1SD-hcp (Li_2ZrCl_6 and $Li_{2.5}ZrCl_{5.5}O_{0.5}$), FD-hcp ($Li_2ZrCl_6$ and $Li_{2.5}ZrCl_{5.5}O_{0.5}$), 1SD-ccp ($Li_2ZrCl_6$ and $Li_{2.25}ZrCl_{5.75}S_{0.25}$) and FD-ccp ($Li_2ZrCl_6$ and $Li_{2.25}ZrCl_{5.75}S_{0.25}$) 1 side-disorder (1SD) means cation disorder occurs asymmetrically near one side of the M2/M3 sites, and M3-site fully disordered (FD) indicates that the M3 sites are entirely filled with metal cations.

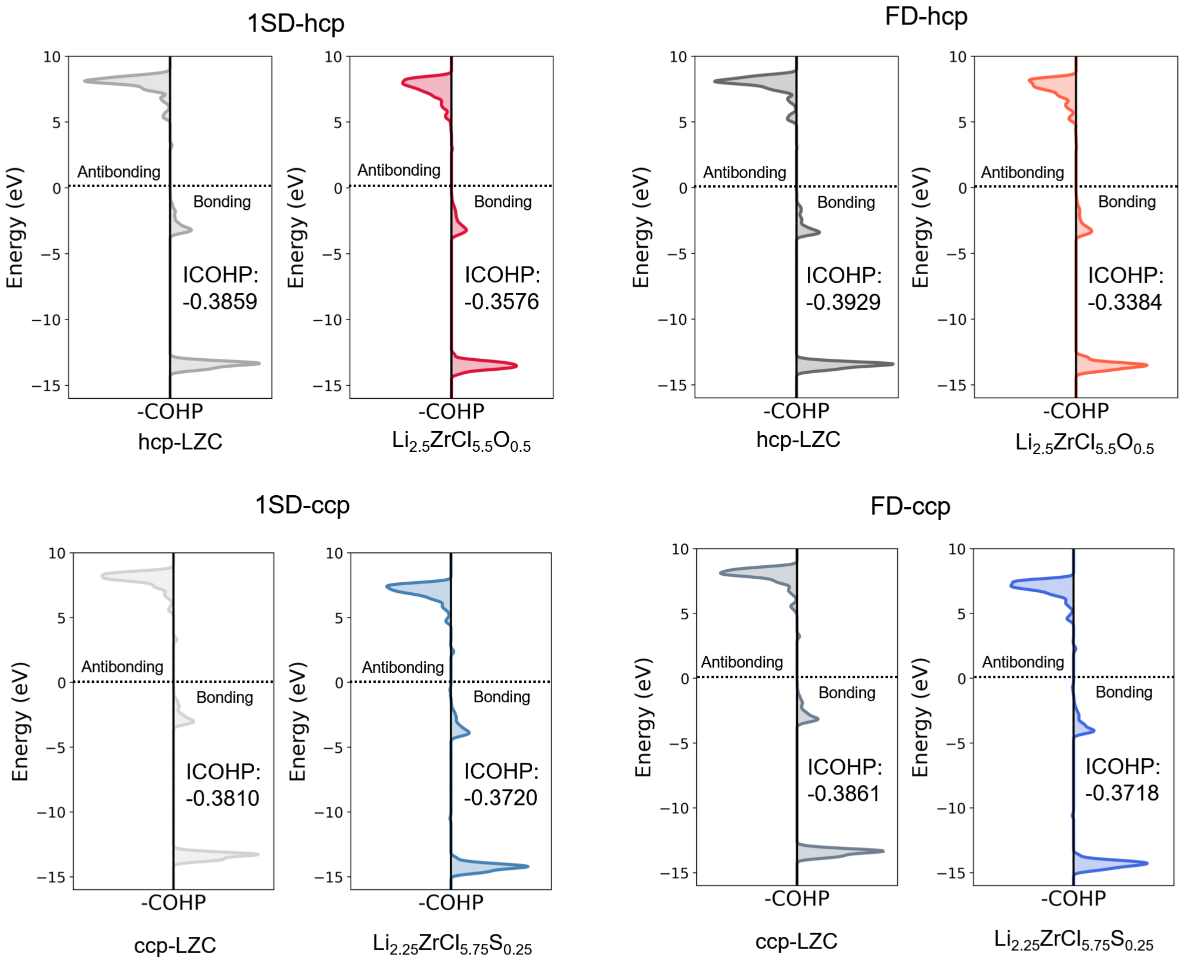

Supplementary Fig. 31. COHP curves with averaged ICOHP values for Li-Cl bonds in 1SD-hcp (Li_2ZrCl_6 and $Li_{2.5}ZrCl_{5.5}O_{0.5}$), FD-hcp ($Li_2ZrCl_6$ and $Li_{2.5}ZrCl_{5.5}O_{0.5}$), 1SD-ccp ($Li_2ZrCl_6$ and $Li_{2.25}ZrCl_{5.75}S_{0.25}$) and FD-ccp ($Li_2ZrCl_6$ and $Li_{2.25}ZrCl_{5.75}S_{0.25}$). 1 side-disorder (1SD) means cation disorder occurs asymmetrically near one side of the M2/M3 sites, and M3-site fully disordered (FD) indicates that the M3 sites are entirely filled with metal cations.

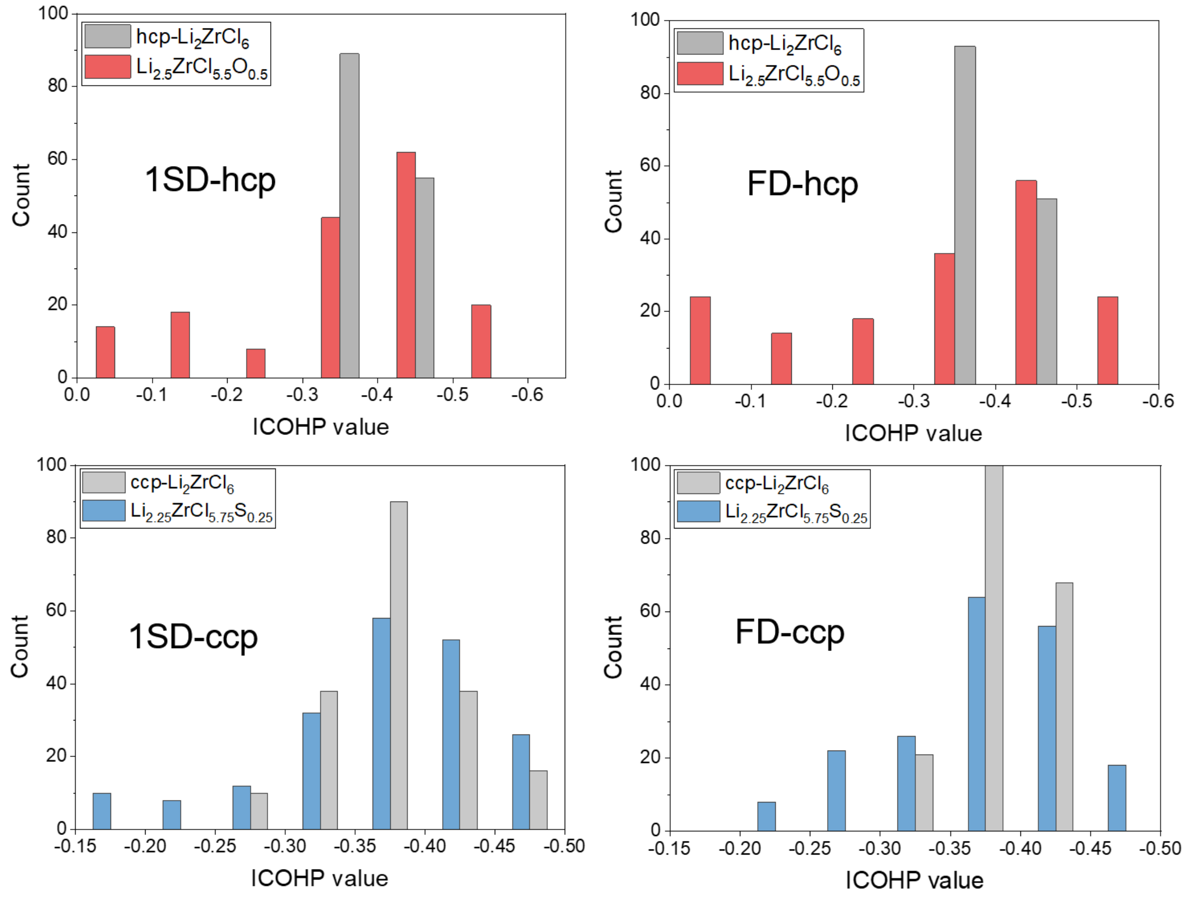

Supplementary Fig. 32. Histograms for distribution of ICOHP values for Li-Cl bonds in 1SD-hcp (Li_2ZrCl_6 and $Li_{2.5}ZrCl_{5.5}O_{0.5}$), FD-hcp ($Li_2ZrCl_6$ and $Li_{2.5}ZrCl_{5.5}O_{0.5}$), 1SD-ccp ($Li_2ZrCl_6$ and $Li_{2.25}ZrCl_{5.75}S_{0.25}$) and FD-ccp ($Li_2ZrCl_6$ and $Li_{2.25}ZrCl_{5.75}S_{0.25}$). 1 side-disorder (1SD) means cation disorder occurs asymmetrically near one side of the M2/M3 sites, and M3-site fully disordered (FD) indicates that the M3 sites are entirely filled with metal cations.

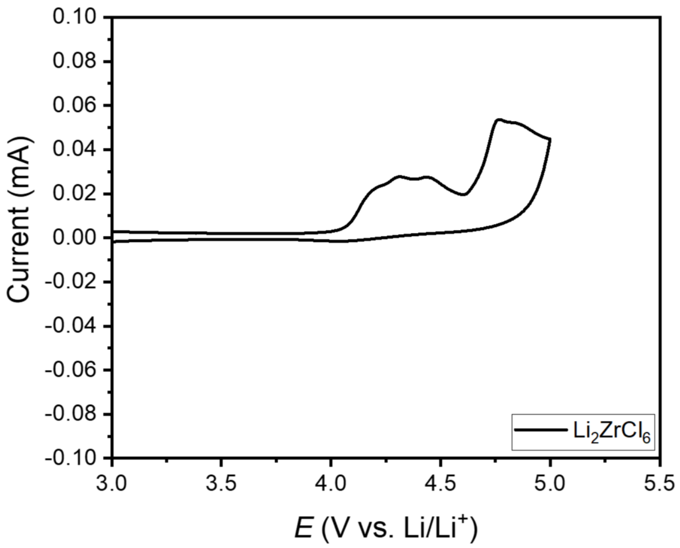

Supplementary Fig. 33. CV curve for hcp-Li_2ZrCl_6 in (Li–In)|LPSC|LZC|(LZC-carbon) cell from 3.0 to 5.0 V (vs. Li/Li^+) at 0.1 mV s^{-1} and 25 °C. The weight ratio of SE:carbon is 7:3.

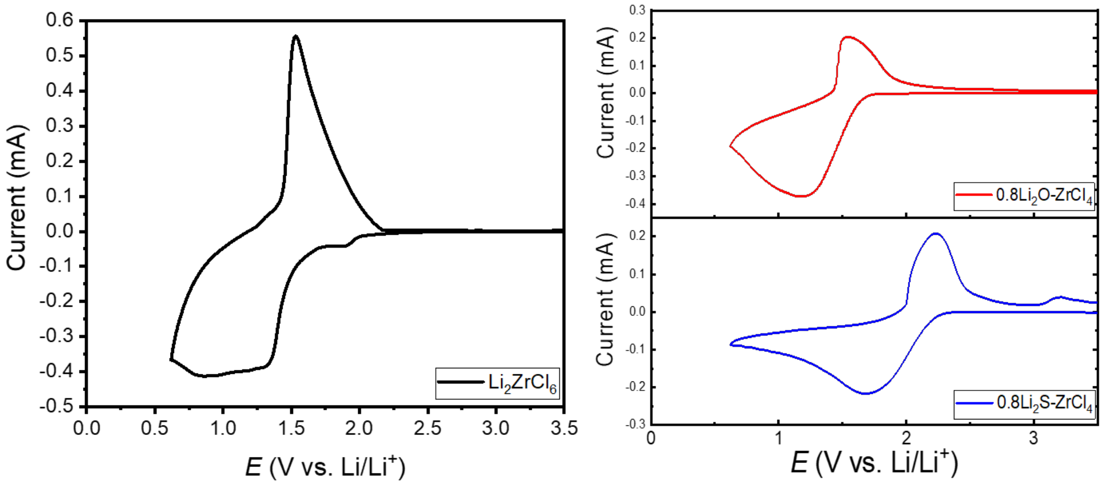

Supplementary Fig. 34. CV curves for hcp-Li_2ZrCl_6 (left), $0.8Li_2O$–$ZrCl_4$ (right-top), $0.8Li_2S$–$ZrCl_4$ (right-bottom) in (Li–In)|LPSC|SE|(SE-carbon) cells from 3.5 to 0.5 V (vs. Li^+/Li) at 0.1 mV s^{-1} and 25 °C. The weight ratio of SE:carbon is 7:3.

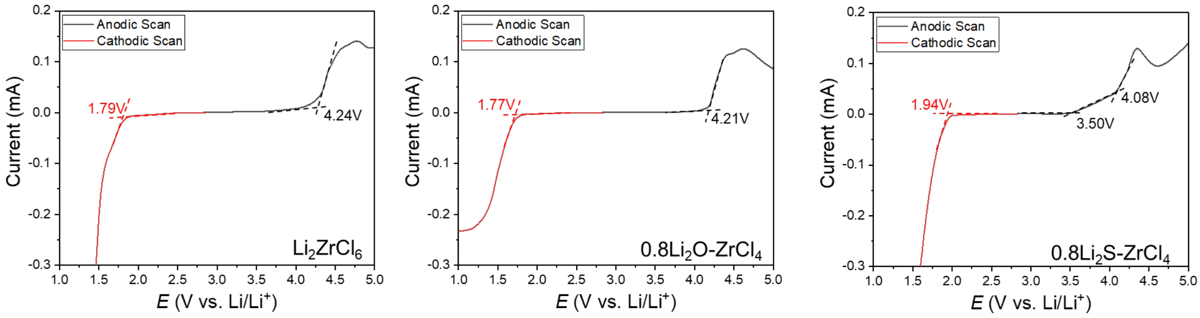

Supplementary Fig. 35. Linear sweep voltammetry (LSV) curves for hcp-Li_2ZrCl_6 (left), 0.8Li_2O–$ZrCl_4$ (middle), 0.8Li_2S–$ZrCl_4$ (right) in (Li–In)|LPSC|SE|(SE-carbon) cells at 0.1 mV s^{-1} and 25 °C. The weight ratio of SE:carbon is 7:3.

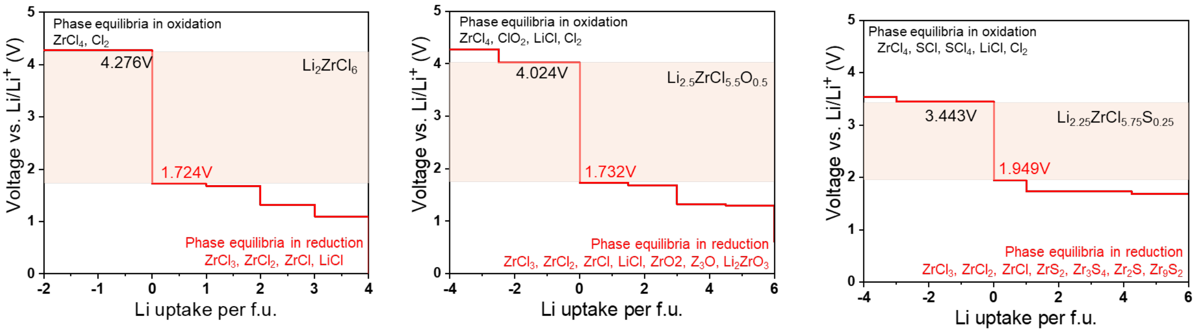

Supplementary Fig. 36. Intrinsic stability window and phase equilibria of hcp-Li_2ZrCl_6, $Li_{2.5}ZrCl_{5.5}O_{0.5}$ and $Li_{2.25}ZrCl_{5.75}S_{0.25}$.

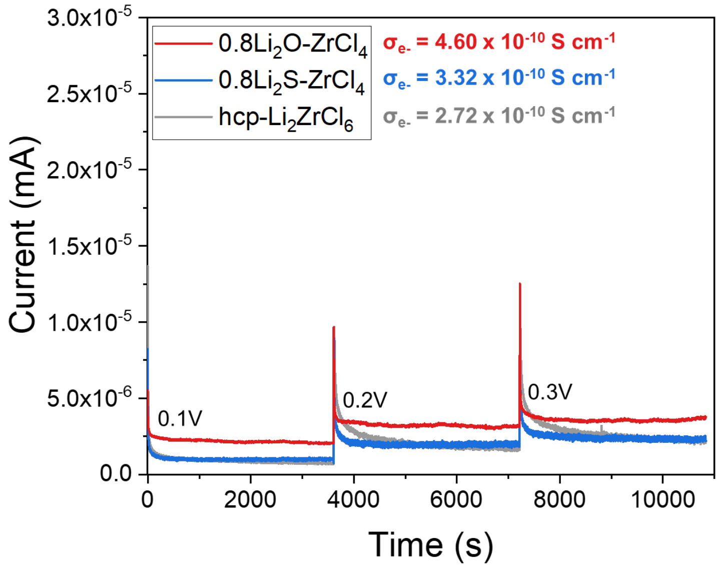

Supplementary Fig. 37. DC measurement for electronic conductivity of $0.8Li_2O–ZrCl_4$, $0.8Li_2S–ZrCl_4$ and hcp-Li_2ZrCl_6.

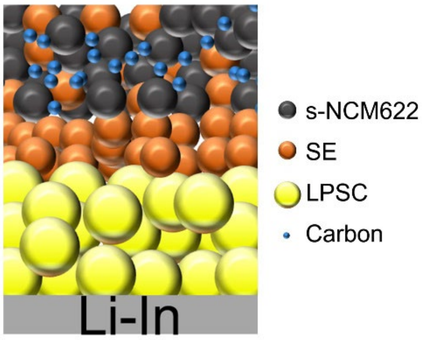

Supplementary Fig. 38. Configurations of all-solid-state batteries with catholytes prepared to test the electrochemical performance of $0.8Li_2A–ZrCl_4$ (A = O, S) and hcp-Li_2ZrCl_6. LPSC, SE, and s-NCM622 means Li_6PS_5Cl, solid electrolyte and single crystalline $LiNi_{0.6}Co_{0.2}Mn_{0.2}O_2$, respectively.

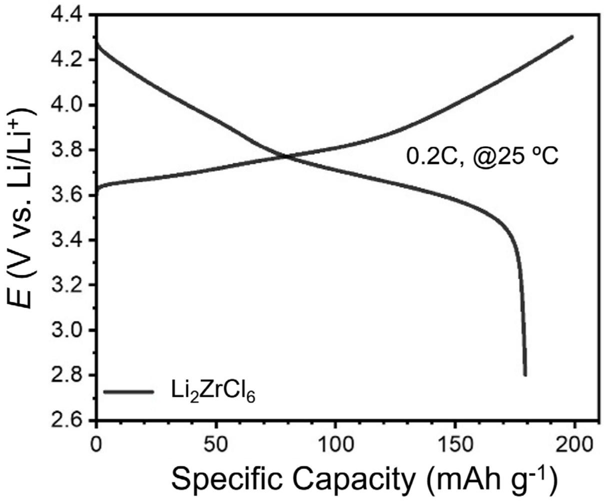

Supplementary Fig. 39. First-cycle charge–discharge voltage profiles for s-NCM622 electrode with Li_2ZrCl_6 (4.3–2.8 V vs. Li/Li^+) at 36 mA g^{-1} (0.2 C) and 25 °C (initial discharge capacity = 179.5 mAh $g^{-1}$and initial coulombic efficiency = 87.8%).

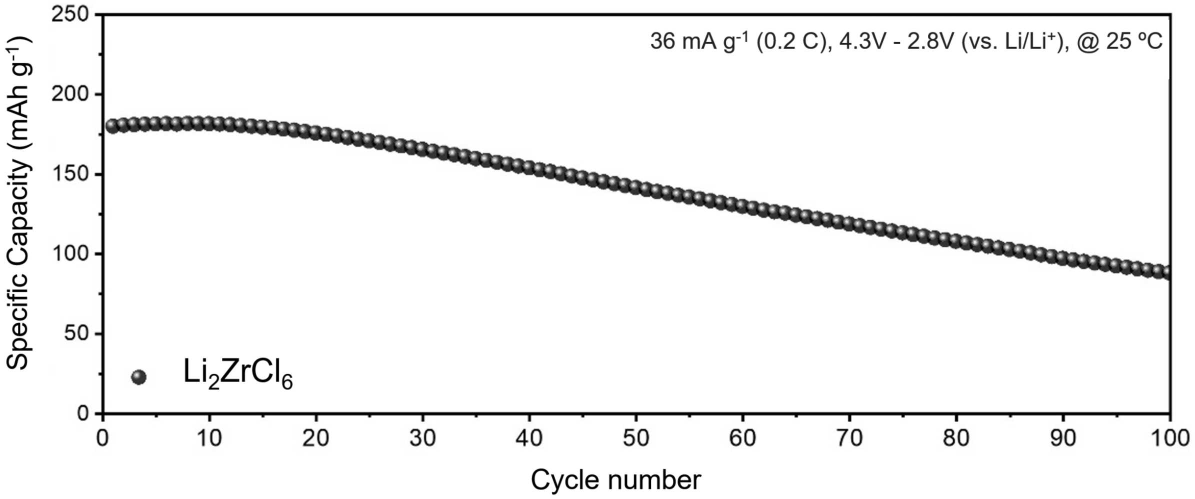

Supplementary Fig. 40. Cycling performance at 36 mA g^{-1} (0.2 C) with Li_2ZrCl_6 at 25℃.

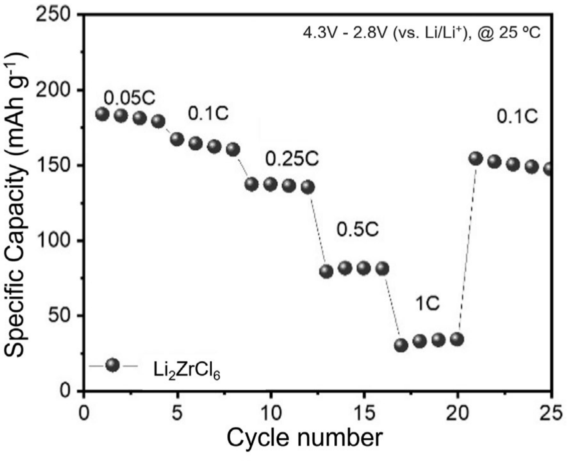

Supplementary Fig. 41. Rate performance at different C-rates (1C = 180 mA g^{-1}) at 25 °C.

2. Supplementary Tables and Supplementary Text

Supplementary Table 1. PDF fitting results of $0.6Li_2O–ZrCl_4$ in PDF short range region (1.5–4.1 Å). Lattice parameters are less convincing due to the fitting range is shorter than lattice parameters.

<table>
<tr><td colspan="4">Crystal system</td><td colspan="4">Trigonal</td></tr>
<tr><td colspan="4">Space group</td><td colspan="4">$P\bar{3}m1$ (164)</td></tr>
<tr><td colspan="4">Lattice parameter</td><td colspan="4">a = b = 11.3814, c = 5.99388</td></tr>
<tr><td colspan="4">Atomic site of hcp-Li_2ZrCl_6 in $0.6Li_2O–ZrCl_4$</td><td rowspan="2">u_{iso} (Å^2)</td><td rowspan="2">Occ.</td><td rowspan="2">Phase fraction (%)</td><td rowspan="2">R_w (%)</td></tr>
<tr><td>Atom</td><td>x</td><td>y</td><td>z</td></tr>
<tr><td>Li1</td><td>0.3397</td><td>0</td><td>0</td><td>0.04</td><td>0.75</td><td rowspan="8">64.6</td><td rowspan="8">10.2</td></tr>
<tr><td>Li2</td><td>0.3397</td><td>0</td><td>0.5</td><td>0.04</td><td>0.25</td></tr>
<tr><td>Zr1</td><td>0</td><td>0</td><td>0</td><td>0.0028</td><td>1.0</td></tr>
<tr><td>Zr2</td><td>0.3333</td><td>0.6667</td><td>0.5382</td><td>0.0028</td><td>0.5</td></tr>
<tr><td>Zr3</td><td>0.3333</td><td>0.6667</td><td>0.0229</td><td>0.0028</td><td>0.5</td></tr>
<tr><td>Cl1</td><td>0.1234</td><td>-0.1234</td><td>0.7504</td><td>0.0042</td><td>1.0</td></tr>
<tr><td>Cl2</td><td>0.2413</td><td>-0.2413</td><td>0.2496</td><td>0.0042</td><td>1.0</td></tr>
<tr><td>Cl3</td><td>0.4411</td><td>-0.4411</td><td>0.7701</td><td>0.0042</td><td>1.0</td></tr>
<tr><td colspan="4">Crystal system</td><td colspan="4">Monoclinic</td></tr>
<tr><td colspan="4">Space group</td><td colspan="4">$P\,1\,2_1/c\,1$ (14)</td></tr>
<tr><td colspan="4">Lattice parameter</td><td colspan="4">a = 5.37439, b = 5.81922, c = 5.5839</td></tr>
<tr><td colspan="4">Atomic site of ZrO_2 in $0.6Li_2O–ZrCl_4$</td><td rowspan="2">u_{iso} (Å^2)</td><td rowspan="2">Occ.</td><td rowspan="2">Phase fraction (%)</td><td rowspan="2">R_w (%)</td></tr>
<tr><td>Atom</td><td>x</td><td>y</td><td>z</td></tr>
<tr><td>Zr1</td><td>0.3259</td><td>0.0495</td><td>0.2036</td><td>0.0028</td><td>1.0</td><td rowspan="3">35.4</td><td rowspan="3">10.2</td></tr>
<tr><td>O1</td><td>0.0312</td><td>0.2787</td><td>0.4327</td><td>0.0062</td><td>1.0</td></tr>
<tr><td>O2</td><td>0.4383</td><td>0.7739</td><td>0.4343</td><td>0.0062</td><td>1.0</td></tr>
</table>

Supplementary Table 2. PDF fitting results of $0.6Li_2O–ZrCl_4$ in PDF long range region (15.0–30.0 Å).

Crystal system				Trigonal		
Space group				$P\bar{3}m1$ (164)		
Lattice parameter				a = b = 10.9701, c = 5.9372		
Atomic site of hcp-Li_2ZrCl_6 in $0.6Li_2O–ZrCl_4$				u_{iso} (Å^2)	Occ.	R_w (%)
Atom	x	y	z			
Li1	0.3397	0	0	0.03	0.75	13.8
Li2	0.3397	0	0.5	0.03	0.25	
Zr1	0	0	0	0.0137	1.0	
Zr2	0.3333	0.6666	0.5330	0.0137	0.49	
Zr3	0.3333	0.6666	0.0122	0.0137	0.51	
Cl1	0.1071	-0.1081	0.7619	0.0271	1.0	
Cl2	0.2265	-0.2265	0.2708	0.0271	1.0	
Cl3	0.4407	-0.4407	0.7470	0.0271	1.0	

Supplementary Table 3. PDF fitting results of $0.8Li_2O–ZrCl_4$ in PDF short range region (1.5–4.1 Å). Lattice parameters are less convincing due to the fitting range is shorter than lattice parameters.

Crystal system				Trigonal			
Space group				$P\bar{3}m1$ (164)			
Lattice parameter				a = b = 11.3368, c= 5.98683			
Atomic site of hcp-Li_2ZrCl_6 in $0.8Li_2O–ZrCl_4$				u_{iso} (Å^2)	Occ.	Phase fraction (%)	R_w (%)
Atom	x	y	z				
Li1	0.3397	0	0	0.04	0.75	65.1	11.0
Li2	0.3397	0	0.5	0.04	0.25		
Zr1	0	0	0	0.0018	1.0		
Zr2	0.3333	0.6667	0.5401	0.0018	0.5		
Zr3	0.3333	0.6667	0.0267	0.0018	0.5		
Cl1	0.1223	-0.1223	0.7535	0.0019	1.0		
Cl2	0.2395	-0.2395	0.2465	0.0019	1.0		
Cl3	0.4419	-0.4419	0.7729	0.0019	1.0		
Crystal system				Monoclinic			
Space group				P 1 2_1/c 1 (14)			
Lattice parameter				a = 5.3869, b = 5.8175, c = 5.55517			
Atomic site of ZrO_2 in $0.8Li_2O–ZrCl_4$				u_{iso} (Å^2)	Occ.	Phase fraction (%)	R_w (%)
Atom	x	y	z				
Zr1	0.3272	0.0516	0.1985	0.0018	1.0	34.9	11.0
O1	0.0513	0.2901	0.4329	0.0029	1.0		
O2	0.4650	0.7741	0.4345	0.0029	1.0		

Supplementary Table 4. PDF fitting results of $0.8Li_2O–ZrCl_4$ in PDF long range region (15.0–30.0 Å).

Crystal system				Trigonal		
Space group				$P\bar{3}m1$ (164)		
Lattice parameter				a = b = 10.9654, c = 5.9324		
Atomic site of hcp-Li_2ZrCl_6 in $0.8Li_2O–ZrCl_4$				u_{iso} (Å^2)	Occ.	R_w (%)
Atom	x	y	z			
Li1	0.3397	0	0	0.03	0.75	15.1
Li2	0.3397	0	0.5	0.03	0.25	
Zr1	0	0	0	0.0125	1.0	
Zr2	0.3333	0.6666	0.5329	0.0125	0.51	
Zr3	0.3333	0.6666	0.0134	0.0125	0.49	
Cl1	0.1082	-0.1082	0.7632	0.0253	1.0	
Cl2	0.2267	-0.2267	0.2717	0.0253	1.0	
Cl3	0.4397	-0.4397	0.7474	0.0253	1.0	

Supplementary Table 5. PDF fitting results of $1.0Li_2O–ZrCl_4$ in PDF short range region (1.5–4.1 Å). Lattice parameters are less convincing due to the fitting range is shorter than lattice parameters.

Crystal system				Trigonal			
Space group				$P\bar{3}m1$ (164)			
Lattice parameter				a = b = 11.3095, c= 5.96056			
Atomic site of hcp-Li_2ZrCl_6 in $1.0Li_2O–ZrCl_4$				u_{iso} (Å^2)	Occ.	Phase fraction (%)	R_w (%)
Atom	x	y	z				
Li1	0.3397	0	0	0.04	0.75	49.9	20.4
Li2	0.3397	0	0.5	0.04	0.25		
Zr1	0	0	0	0.0021	1.0		
Zr2	0.3333	0.6667	0.5456	0.0021	0.5		
Zr3	0.3333	0.6667	0.0238	0.0021	0.5		
Cl1	0.1173	-0.1173	0.7570	0.0049	1.0		
Cl2	0.2339	-0.2339	0.2430	0.0049	1.0		
Cl3	0.4414	-0.4414	0.7700	0.0049	1.0		
Crystal system				Monoclinic			
Space group				P 1 2_1/c 1 (14)			
Lattice parameter				a = 5.53951, b = 5.82518, c = 5.52475			
Atomic site of ZrO_2 in $1.0Li_2O–ZrCl_4$				u_{iso} (Å^2)	Occ.	Phase fraction (%)	R_w (%)
Atom	x	y	z				
Zr1	0.3297	0.0525	0.1926	0.0021	1.0	50.1	20.4
O1	0.0428	0.2674	0.4016	0.0073	1.0		
O2	0.4454	0.7395	0.4270	0.0073	1.0		

Supplementary Table 6. PDF fitting results of $1.0Li_2O–ZrCl_4$ in PDF long range region (15.0–30.0 Å). The fitting results are less convincing due to significant high R_w factor.

Crystal system				Trigonal		
Space group				$P\bar{3}m1$ (164)		
Lattice parameter				a = b = 10.9635, c = 5.9251		
Atomic site of hcp-Li_2ZrCl_6 in $1.0Li_2O–ZrCl_4$				u_{iso} (Å^2)	Occ.	R_w (%)
Atom	x	y	z			
Li1	0.3397	0	0	0.03	0.75	34.0
Li2	0.3397	0	0.5	0.03	0.25	
Zr1	0	0	0	0.011	1.0	
Zr2	0.3333	0.6666	0.5385	0.011	0.64	
Zr3	0.3333	0.6666	0.0008	0.011	0.36	
Cl1	0.1142	-0.1142	0.7632	0.0176	1.0	
Cl2	0.2275	-0.2275	0.2554	0.0176	1.0	
Cl3	0.4350	-0.4350	0.7472	0.0176	1.0	

Supplementary Table 7. EXAFS fitting results of xLi$_2$O–ZrCl$_4$ (x = 0.6, 0.8, 1.0) corresponding to **Supplementary Fig. 6**. a) crystallographic multiplicity (degeneracy of atomic pairs at distance R). b) interatomic distance between absorber and scatterer atoms. c) Debye-Waller factor (mean square disorder of the absorber-scatterer distance). d) edge energy shift parameter. e) Reliability factor. Detailed fitting procedures and definition of N used in the fitting are shown in **Supplementary Text 3.**

Sample	Phase	Shell	Path	N[a]	R (Å)[b]	Range of error(Å)	σ^2 (Å^2)[c]	Range of error(Å^2)	Phase fraction of ZrO_2(%)	Phase fraction of hcp (%)	Range of error(%P)	ΔE(eV)[d]	Range of error(eV)	R-factor (%)[e]
0.6Li$_2$O-ZrCl$_4$	ZrO_2	1st	Zr-O	7	2.030	0.010	0.009	0.001	34.91	65.09	4.41	-2.25	1.25	1.55
		2nd	Zr-Zr	3	3.286	0.040	0.006	0.000						
			Zr-Zr	3	3.426	0.036	0.005	0.001						
			Zr-Zr	3	3.673	0.028	0.005	0.001						
	Li_2ZrCl_6 (hcp)	1st	Zr-Cl	6	2.465	0.010	0.013	0.001						
		2nd	Zr(M2)-Zr(M3)	2	2.764	0.010	0.006	0.000						
0.8Li$_2$O-ZrCl$_4$	ZrO_2	1st	Zr-O	7	2.040	0.009	0.006	0.000	35.16	64.84	4.04	-1.94	1.22	1.78
		2nd	Zr-Zr	3	3.322	0.030	0.005	0.000						
			Zr-Zr	3	3.448	0.031	0.004	0.001						
			Zr-Zr	3	3.693	0.029	0.004	0.001						
	Li_2ZrCl_6 (hcp)	1st	Zr-Cl	6	2.477	0.009	0.012	0.001						
		2nd	Zr(M2)-Zr(M3)	2	2.811	0.009	0.005	0.000						
1.0Li$_2$O-ZrCl$_4$	ZrO_2	1st	Zr-O	7	2.052	0.007	0.009	0.001	52.00	48.00	2.85	-3.17	1.06	1.45
		2nd	Zr-Zr	3	3.336	0.017	0.005	0.000						
			Zr-Zr	3	3.478	0.017	0.004	0.000						
			Zr-Zr	3	3.685	0.010	0.004	0.000						
	Li_2ZrCl_6 (hcp)	1st	Zr-Cl	6	2.483	0.007	0.011	0.001						
		2nd	Zr(M2)-Zr(M3)	2	2.852	0.007	0.005	0.000						

* E_0 (eV) = 18008.10, S_0^2 = 0.75. All spectra were Fourier transformed in k-range of 3.1 to 12.8 Å^{-1} and fitted in R range of 1.35 to 3.5 Å

Supplementary Table 8. PDF fitting results of $0.6Li_2S–ZrCl_4$ in PDF short range region (1.5–4.1 Å). Lattice parameters are less convincing due to the fitting range is shorter than lattice parameters.

Crystal system				Monoclinic			
Space group				$C2/m$ (12)			
Lattice parameter				a = 6.23829, b = 11.836, c= 6.44117			
Atomic site of ccp-Li_2ZrCl_6 in $0.6Li_2S–ZrCl_4$				u_{iso} (Å^2)	Occ.	Phase fraction (%)	R_w (%)
Atom	x	y	z				
Li1	0.5	0	0.5	0.04	0.15	64.2	12.9
Li2	0	0.18	0.5	0.04	0.1		
Li3	0	0.325	0	0.04	0.86		
Zr1	0	0	0	0.0085	0.77		
Zr2	0	0.3250	0	0.0085	0.06		
Zr3	0.5	0	0.5	0.0085	0.1		
Cl1	0.2532	0	0.7677	0.0099	1.0		
Cl2	0.2533	0.1462	0.2164	0.0099	1.0		
Crystal system				Trigonal			
Space group				$P\bar{3}m1$ (164)			
Lattice parameter				a = b = 3.68882, c = 5.78867			
Atomic site of ZrS_2 in $0.6Li_2S–ZrCl_4$				u_{iso} (Å^2)	Occ.	Phase fraction (%)	R_w (%)
Atom	x	y	z				
Zr1	0	0	0	0.0086	1.0	35.8	12.9
S1	0.3333	0.6667	0.25	0.0099	1.0		

Supplementary Table 9. PDF fitting results of $0.6Li_2S-ZrCl_4$ in PDF long range region (15.0–30.0 Å).

Crystal system				Monoclinic		
Space group				$C2/m$ (12)		
Lattice parameter				a = 6.41578, b = 11.0744, c = 6.3875		
Atomic site of ccp-Li_2ZrCl_6 in $0.6Li_2S-ZrCl_4$				u_{iso} (Å^2)	Occ.	R_w (%)
Atom	x	y	z			
Li1	0.5	0	0.5	0.04	0.15	16.4
Li2	0	0.18	0.5	0.04	0.1	
Li3	0	0.325	0	0.04	0.86	
Zr1	0	0	0	0.0273	0.77	
Zr2	0	0.3188	0	0.0273	0.06	
Zr3	0.5	0	0.5	0.0273	0.1	
Cl1	0.2417	0	0.7522	0.0352	1.0	
Cl2	0.2462	0.1647	0.2481	0.0352	1.0	

Supplementary Table 10. PDF fitting results of 0.8Li_2S–$ZrCl_4$ in PDF short range region (1.5–4.1 Å). Lattice parameters are less convincing due to the fitting range is shorter than lattice parameters.

Crystal system				Monoclinic			
Space group				$C2/m$ (12)			
Lattice parameter				a = 6.32927, b = 11.152, c= 6.4559			
Atomic site of ccp-Li_2ZrCl_6 in 0.8Li_2S–$ZrCl_4$				u_{iso} ($Å^2$)	Occ.	Phase fraction (%)	R_w (%)
Atom	x	y	z				
Li1	0.5	0	0.5	0.04	0.15	68.1	10.8
Li2	0	0.18	0.5	0.04	0.1		
Li3	0	0.325	0	0.04	0.86		
Zr1	0	0	0	0.0126	0.77		
Zr2	0	0.2470	0	0.0126	0.06		
Zr3	0.5	0	0.5	0.0126	0.1		
Cl1	0.2343	0	0.7421	0.0116	1.0		
Cl2	0.2777	0.1441	0.2305	0.0116	1.0		
Crystal system				Trigonal			
Space group				$P\bar{3}m1$ (164)			
Lattice parameter				a = b = 3.67977, c = 5.77874			
Atomic site of ZrS_2 in 0.8Li_2S–$ZrCl_4$				u_{iso} ($Å^2$)	Occ.	Phase fraction (%)	R_w (%)
Atom	x	y	z				
Zr1	0	0	0	0.0126	1.0	31.9	10.8
S1	0.3333	0.6667	0.25	0.0117	1.0		

Supplementary Table 11. PDF fitting results of $0.8Li_2S–ZrCl_4$ in PDF long range region (15.0–30.0 Å).

Crystal system					Monoclinic	
Space group					$C2/m$ (12)	
Lattice parameter					a = 6.4039, b = 11.0639, c = 6.3850	
Atomic site of ccp-Li_2ZrCl_6 in $0.8Li_2S–ZrCl_4$				u_{iso} (Å^2)	Occ.	R_w (%)
Atom	x	y	z			
Li1	0.5	0	0.5	0.04	0.15	15.3
Li2	0	0.18	0.5	0.04	0.1	
Li3	0	0.325	0	0.04	0.86	
Zr1	0	0	0	0.0318	0.77	
Zr2	0	0.3158	0	0.0318	0.06	
Zr3	0.5	0	0.5	0.0318	0.1	
Cl1	0.2432	0	0.7508	0.0331	1.0	
Cl2	0.2426	0.1653	0.249	0.0331	1.0	

Supplementary Table 12. PDF fitting results of $1.0Li_2S–ZrCl_4$ in PDF short range region (1.5–4.1 Å). Lattice parameters are less convincing due to the fitting range is shorter than lattice parameters.

Crystal system				Monoclinic			
Space group				$C2/m$ (12)			
Lattice parameter				a = 6.29498, b = 11.4343, c= 6.23637			
Atomic site of ccp-Li_2ZrCl_6 in $1.0Li_2S–ZrCl_4$				u_{iso} (Å^2)	Occ.	Phase fraction (%)	R_w (%)
Atom	x	y	z				
Li1	0.5	0	0.5	0.04	0.15	71.4	15.3
Li2	0	0.18	0.5	0.04	0.1		
Li3	0	0.325	0	0.04	0.86		
Zr1	0	0	0	0.0096	0.77		
Zr2	0	0.2431	0	0.0096	0.06		
Zr3	0.5	0	0.5	0.0096	0.1		
Cl1	0.2323	0	0.7463	0.0132	1.0		
Cl2	0.2751	0.1495	0.2371	0.0132	1.0		
Crystal system				Trigonal			
Space group				$P\bar{3}m1$ (164)			
Lattice parameter				a = b = 3.75216, c = 6.02956			
Atomic site of ZrS_2 in $1.0Li_2S–ZrCl_4$				u_{iso} (Å^2)	Occ.	Phase fraction (%)	R_w (%)
Atom	x	y	z				
Zr1	0	0	0	0.0096	1.0	28.6	15.3
S1	0.3333	0.6667	0.25	0.0132	1.0		

Supplementary Table 13. PDF fitting results of $1.0Li_2S–ZrCl_4$ in PDF long range region (15.0–30.0 Å).

Crystal system				Monoclinic		
Space group				$C2/m$ (12)		
Lattice parameter				a = 6.36994, b = 11.0442, c = 6.3762		
Atomic site of ccp-Li_2ZrCl_6 in $1.0Li_2S–ZrCl_4$				u_{iso} (Å^2)	Occ.	R_w (%)
Atom	x	y	z			
Li1	0.5	0	0.5	0.04	0.15	17.6
Li2	0	0.18	0.5	0.04	0.1	
Li3	0	0.325	0	0.04	0.86	
Zr1	0	0	0	0.0322	0.77	
Zr2	0	0.2489	0	0.0322	0.06	
Zr3	0.5	0	0.5	0.0322	0.1	
Cl1	0.2413	0	0.7546	0.0239	1.0	
Cl2	0.2444	0.1620	0.2488	0.0239	1.0	

Supplementary Table 14. EXAFS fitting results of xLi$_2$S–ZrCl$_4$ (x = 0.6, 0.8, 1.0) corresponding to **Supplementary Fig. 12**. a) crystallographic multiplicity (degeneracy of atomic pairs at distance R). b) interatomic distance between absorber and scatterer atoms. c) Debye-Waller factor (mean square disorder of the absorber-scatterer distance). d) edge energy shift parameter. e) Reliability factor. Detailed fitting procedures and definition of N used in the fitting are shown in **Supplementary Text 3.**

Sample	Phase	Shell	Path	N[a]	R (Å)[b]	Range of error(Å)	σ^2 (Å^2)[c]	Range of error(Å^2)	Phase fraction of ZrS$_2$(%)	Phase fraction of ccp (%)	Range of error(% P)	ΔE (eV)[d]	Range of error(eV)	R-factor (%)[e]
0.6Li$_2$S-ZrCl$_4$	ZrS$_2$	1st	Zr-S	6	2.550	0.023	0.008	0.001	32.06	67.94	21.40	-1.62	2.32	1.95
		2nd	Zr-Zr	6	3.741	0.050	0.013	0.001						
	Li$_2$ZrCl$_6$ (ccp)	1st	Zr-Cl	6	2.453	0.023	0.008	0.001						
		2nd	Zr(2a)-Zr(4g)	2	3.609	0.050	0.011	0.001						
			Zr(2a)-Zr(2d)	2	3.663	0.050	0.011	0.001						
			Zr(2a)-Zr(4g)	4	3.755	0.050	0.011	0.001						
0.8Li$_2$S-ZrCl$_4$	ZrS$_2$	1st	Zr-S	6	2.568	0.020	0.007	0.001	29.32	70.68	19.20	-0.61	2.08	1.68
		2nd	Zr-Zr	6	3.739	0.045	0.012	0.001						
	Li$_2$ZrCl$_6$ (ccp)	1st	Zr-Cl	6	2.471	0.020	0.007	0.001						
		2nd	Zr(2a)-Zr(4g)	2	3.649	0.045	0.010	0.001						
			Zr(2a)-Zr(2d)	2	3.703	0.045	0.010	0.001						
			Zr(2a)-Zr(4g)	4	3.795	0.045	0.010	0.001						
1.0Li$_2$S-ZrCl$_4$	ZrS$_2$	1st	Zr-S	6	2.618	0.015	0.009	0.001	25.72	74.28	12.01	1.40	1.84	1.56
		2nd	Zr-Zr	6	3.753	0.031	0.010	0.001						
	Li$_2$ZrCl$_6$ (ccp)	1st	Zr-Cl	6	2.510	0.015	0.009	0.001						
		2nd	Zr(2a)-Zr(4g)	2	3.663	0.031	0.010	0.001						
			Zr(2a)-Zr(2d)	2	3.717	0.031	0.010	0.001						
			Zr(2a)-Zr(4g)	4	3.809	0.031	0.010	0.001						

* E_0 (eV) = 18008.10, S_0^2 = 0.75. All spectra were Fourier transformed in k-range of 3.8 to 11.2 Å^{-1} and fitted in R range of 1.7 to 3.7 Å

Supplementary Text 1. The presence of monoclinic ZrO_2
We conducted PDF calculations to identify whether ZrO_2 is cubic or monoclinic. The PDF calculations clearly revealed characteristic features of monoclinic ZrO_2 in the $0.8Li_2O$-$ZrCl_4$ composition, confirming the presence of monoclinic ZrO_2 **(Supplementary Fig. 3)**.

Supplementary Text 2. Phase ratio of PDF and EXAFS fitting model for Supplementary Fig. 4,6,10,12 and Supplementary Table 1-14.
Given that overwhelming scattering power from Zr in ZrO_2, ZrS_2, and the hcp, ccp phases compared to that of Li_2O and LiCl, the latter two were excluded in PDF and EXAFS fitting to reduce parameter thereby securing fitting reliability. Additionally, anion exchange was not considered in PDF and EXAFS refinement due to the following reasons:

1. A slight first shell intensity reduction was observed in $x$$Li_2O$–$ZrCl_4$ (x = 0.6, 0.8, 1.0) due to anion exchange in hcp-Li_2ZrCl_6. **(Fig. 2a)** However, as this reduction is marginal, the fitting for hcp-Li_2ZrCl_6 was performed considering only the Zr-Cl coordination and excluding small amount of Zr-O bond.
2. The isoelectronic feature of Cl and S (18 electrons) make it heavily challenging to refine substitution fraction due to similar X-ray scattering power.

We constructed EXAFS fitting model for ZrO_2 which nine coordinated Zr-Zr distances were divided into three subsets to capture Zr-Zr correlations in amorphous ZrO_2. Continually, site disorder at M2-M3 in the hcp phase was fixed at 50% based on our PDF fitting results, which demonstrate M2-M3 site disorder is near 0.5. (**Supplementary Tables 2, 4, 6)**

Although Li_2O and Li_2S were excluded from the refinements, the obtained fractions of ZrO_2 vs. hcp phase and ZrS_2 vs. ccp phase by both PDF and EXAFS fitting closely matched with computational predictions (**Supplementary Tables 1-14**) This agreement strongly suggests the absence of residual precursor or unreacted Li_2ZrCl_6 phases in the $x$$Li_2O$–$ZrCl_4$ and $x$$Li_2S$–$ZrCl_4$ systems. Therefore, we suggest that the phase fractions of Li_2O and Li_2S, as well as the amount of O and S substitution in the hcp and ccp phases, closely align with computationally predicted values.

Supplementary Text 3. EXAFS fitting model of Li_2O-$ZrCl_4$ and Li_2S-$ZrCl_4$.
For EXAFS fitting, the crystallographic information of hcp- Li_2ZrCl_6 was taken from the Supporting Information (Table S1) of ref [38] in manuscript. Additionally, the model of ccp-Li_2ZrCl_6 was constructed by adopting Li_3YCl_6 (ICSD 29963), replacing Y with Zr, which has comparable atomic mass and structure. In hcp-Li_2ZrCl_6, occupancies were set to 1.0 for M1 and 0.5 for M2/M3, based on PDF refinements yielding ≈ 0.5 for the latter sites. Similarly, the occupancies of Zr at the ccp-Li_2ZrCl_6 2a, 4g, and 2d sites were fixed at 0.8, 0.05, and 0.1, respectively to simplify the model.

N in **Supplementary Table 7** and **Supplementary Table 14** does not correspond to all actual coordination present in the structure but rather reflects a crystallographic multiplicity (degeneracy of atomic pairs at distance R) listed in the table. In the EXAFS scattering power as shown in below theoretical EXAFS scattering power equation, N_j acts as an effective coordination parameter that scales the scattering amplitude, and in our fitting, it inherently includes contributions from multiplicities and site occupancies.

$$\chi(k) = \sum_j \frac{N_j S_0^2 F_j(k)}{kR_j^2} e^{-2R_j/\lambda(k)} e^{-2\sigma_j^2 k^2} \sin\left(2kR_j + \delta_j(k)\right) \quad (4)$$

Where: N_j: coordination number, S_0^2: amplitude reduction factor, $F_j(k)$: effective back-scattering amplitude, k: photoelectron wave number, R_j: effective half path length, $\lambda(k)$: inelastic mean free path σ_j^2: Debye-Waller factor.

In this context, N_j is obtained by occupancy × multiplicity (N as shown in Supplementary Table 7 and Supplementary Table 14) as an effective coordination parameter that incorporates both site occupancies and the corresponding coordination environment. In EXAFS fitting model, each path amplitude is weighted by the site occupancy and multiplicity (e.g., N_j of Zr(2a)-Zr(4g) = N (multiplicity) × 0.8 (occupancy of 2a site) × 0.05 (occupancy of 4g site) = 0.08).

We included Zr-Cl and Zr-Zr paths within 1.35 to 3.5 Å considering these occupancies for the O-doped samples. For the S-doped samples, Zr-Cl, Zr(2a)-Zr(4g), and Zr(2a)-Zr(2d) paths within 1.7 to 3.7 Å were included, while the Zr(4g)-Zr(2d) path was excluded based on theoretical EXAFS scattering power.

As scattering amplitude is proportional to the coordination number and site occupancy, we weight each path amplitude by the corresponding occupancy. All Zr–Zr paths involve identical scatterers- Zr - the element-dependent component of *F(k)* is constant across these paths. The table below lists the pairwise products of Zr-site occupancies used as scattering power weights for the respective paths.

Path	Relative scattering power
Zr(2a)-Zr(4g)	0.8 × 0.05=0.04
Zr(2a)-Zr(2d)	0.8 × 0.1=0.08
Zr(4g)-Zr(2d)	0.05 × 0.1=0.005

Accounting for the three Zr-site occupancies in ccp-Li_2ZrCl_6, the scattering power weight of the Zr(4g)-Zr(2d) pair amounts to ~4.17% of the combined Zr(2a)-Zr(4g) and Zr(2a)-Zr(2d) contributions ($\frac{[Zr(4g)-Zr(2d)\ path]}{[Zr(2a)-Zr(4g)\ path]+[Zr(2a)-Zr(2d)\ path]} = \frac{0.005}{0.04+0.08} = 0.0417$). Therefore, considering marginal scattering power between Zr(4g)-Zr(2d) path, we excluded the path from the fitting.

Supplementary Text 4. Amorphous phase

The peaks corresponding to the hcp and ccp phases were well maintained within the PDF G(r) range of 1.5-6 Å for the compositions xLi_2O–$ZrCl_4$ and xLi_2S–$ZrCl_4$ (x = 0.6, 0.8, 1.0) (Fig. 2a, 3a). Given that ~6 Å corresponds approximately to the lattice parameter (interlayer distance) along the c-axis of Li_2ZrCl_6 (6.030 Å for hcp and 6.376 Å for ccp), the sustained intensity of this peak suggests that the long-range periodicity associated with these lattice frameworks is preserved. The consistent peak behavior in this region thus supports the retention of hcp and ccp structural motifs without meaningful evidence of amorphous phase development. Thus, we conclude that there is an only negligible amount of amorphous phase formation in these materials at compositions corresponding to the highest ionic conductivity (x = 0.6, 0.8, 1.0). While structural amorphization in Zr-based oxyhalide has been proposed in prior studies as a mechanism for enhancing ionic conductivity, particularly within their respective systems, our compositions and results suggest a different origin for the observed conductivity. Samples with lower crystallinity (such as $1.0Li_2O$–$ZrCl_4$ and $1.2Li_2O$–$ZrCl_4$) exhibited reduced ionic conductivity compared to the more crystalline $0.8Li_2O$–$ZrCl_4$ composition (**Fig. 1b,c**). This behavior shows that amorphization was not linked to improved ionic transport, suggesting instead that the high ionic

conductivity in our system originates from an optimized crystalline structure rather than from any amorphous contribution. Thus, we conclude that there is only negligible amount of amorphous phase in these materials at compositions corresponding to the highest ionic conductivity (x = 0.6, 0.8, 1.0).

Supplementary Table 15. The average bond length of Zr-anion in hcp-Li_2ZrCl_6, hcp-$Li_{2.5}ZrCl_{5.5}O_{0.5}$, ccp-$Li_2ZrCl_6$, ccp-$Li_{2.25}ZrCl_{5.75}S_{0.25}$, m-$ZrO_2$, and ZrS_2. All the structures were fully relaxed by DFT calculations.

	hcp-Li_2ZrCl_6	$Li_{2.5}ZrCl_{5.5}O_{0.5}$	ccp-Li_2ZrCl_6	$Li_{2.25}ZrCl_{5.75}S_{0.25}$	m-ZrO_2	ZrS_2
Zr-Cl	2.490Å	2.539Å	2.486Å	2.498Å	-	-
Zr-O	-	1.895Å	-	-	2.163Å	-
Zr-S	-	-	-	2.381Å	-	2.571Å

Supplementary Table 16. Energy above the hull and phase equilibria at the hull of hcp-$Li_{2+x}ZrCl_{6-x}O_x$ (x = 0, 0.166, 0.333, 0.5, 0.667, 0.833, and 1).

Composition	E_{hull} (meV atom^{-1})	Phase equilibria
(Li_2ZrCl_6) $Li_{12}Zr_6Cl_{36}$	7	LiCl, $ZrCl_4$
(x = 0.166) $Li_{13}Zr_6Cl_{35}O_1$	24	LiCl, $ZrCl_4$, ZrO_2
(x = 0.333) $Li_{14}Zr_6Cl_{34}O_2$	48	
(x = 0.5) $Li_{15}Zr_6Cl_{33}O_3$	63	
(x = 0.667) $Li_{16}Zr_6Cl_{32}O_4$	70	
(x = 0.833) $Li_{17}Zr_6Cl_{31}O_5$	86	
(x = 1) $Li_{18}Zr_6Cl_{30}O_6$	101	

Supplementary Table 17. Energy above the hull and phase equilibria at the hull of ccp-$Li_{2+x}ZrCl_{6-x}S_x$ (x = 0, 0.125, 0.25, 0.375, 0.5, 0.625, 0.75, 0.875).

Composition	E_{hull} (meV atom^{-1})	Phase equilibria
(Li_2ZrCl_6) $Li_{16}Zr_8Cl_{48}$	2	LiCl, $ZrCl_4$
(x = 0.125) $Li_{17}Zr_8Cl_{47}S_1$	5	
(x = 0.25) $Li_{18}Zr_8Cl_{46}S_2$	11	
(x = 0.375) $Li_{19}Zr_8Cl_{45}S_3$	20	
(x = 0.5) $Li_{20}Zr_8Cl_{44}S_4$	27	LiCl, $ZrCl_4$, ZrS_2
(x = 0.625) $Li_{21}Zr_8Cl_{43}S_5$	36	
(x = 0.75) $Li_{22}Zr_8$ $Cl_{42}S_6$	43	
(x = 0.875) $Li_{23}Zr_8Cl_{41}S_7$	50	

Supplementary Table 18. Various chemical reaction equations and reaction energy (E_{Rxn}) with experimental ionic conductivity of oxygen-source component. E_{rxn} is calculated by DFT energies.

Index	Reaction Equations	E_{Rxn} (eV/atom)	Experimental ionic conductivity (mS cm^{-1})
1	$12Li_2O + 15ZrCl_4$ → $2Li_2O + 5ZrO_2 + 10Li_2ZrCl_6$*	-0.219	1.78
2	$10Li_2O+11ZrCl_4$ → $3ZrO_2 + 8Li_{2.5}ZrCl_{5.5}O_{0.5}$	-0.212	1.42
3	$2Li_2O + 3ZrCl_4$ → $ZrO_2 + 2Li_2ZrCl_6$	-0.219	1.18
4	$4LiCl + 5ZrCl_4 + 2Li_2O$ → $ZrO_2 + 4Li_2ZrCl_6$	-0.115	0.99
5	$2Li_2O + 2ZrCl_4$ → $2LiCl + ZrO_2 + Li_2ZrCl_6$	-0.292	1.33
6	$2Li_2O+ZrCl_4$ → $4LiCl + ZrO_2$	-0.431	~10^{-4}

Supplementary Text 5. Reaction 1 ($x = 0.8$, interfacial anion substitution) vs. Reaction 2 ($x \approx 0.9$, doping all oxygen into the bulk without Li_2O)
The basis for judging that Reaction 2 is impossible and Reaction 1 is possible is as follows. First, Li_2O was observed in the synchrotron XRD pattern (Figure 1c), and the presence of Li_2O indicates that it is difficult for the structure to accommodate oxygen. Second, the E_{hull} in Supplementary Table 16 is relatively high, making direct substitution difficult in environments without oxygen concentration, such as at the interface. Third, if full substitution had occurred in the bulk, the highest conductivity would have been observed at x = 0.9; however, this is not the case. This implies that the composition where Li_2O and ZrO_2 most readily form an hcp-Li_2ZrCl_6 phase with oxygen-substituted interfaces is x=0.8.
Supplementary Text 6. While the stoichiometry $2Li_2O + 5ZrO_2 + 10Li_2ZrCl_6$* is used to represent the dominant phase distribution after reaction, the actual structure involves partial and spatially heterogeneous anion substitution (**Supplementary Fig. 9**), which can be described by the generalized expression $2Li_{2-5x}O_{1-5x}Cl_{5x} + 5ZrO_{2-2y}Cl_{2y} + 10Li_{2+x}ZrCl_{(6-x-y)}O_{(x+y)}$ to reflect local compositional gradients within the nanocomposite.

Supplementary Table 19. Various chemical reaction equations and reaction energy (E_{Rxn}) with experimental ionic conductivity of sulfur-source component. E_{rxn} is calculated by DFT energies.

Index	Reaction Equations	E_{Rxn} (eV/atom)	Experimental ionic conductivity (mS cm^{-1})
7	$2Li_2S + 3ZrCl_4$ → $ZrS_2 + 2Li_2ZrCl_6$	-0.105	0.88
8	$18Li_2S + 23ZrCl_4$ → $7ZrS_2 + 16Li_{2.25}ZrCl_{5.75}S_{0.25}$	-0.112	1.01
9	$2Li_2S+ZrCl_4$ → $4LiCl + ZrS_2$	-0.208	~10^{-3}

Supplementary Text 7. Reaction 7 ($x \approx 0.67$, interfacial substitution) vs. Reaction 8 ($x \approx 0.78$, conditions required for doping all sulfur into the bulk)

Unlike oxygen, sulfur appears to undergo bulk substitution. As explained in the text, no ccp phase was observed among the Zr-based solid electrolytes synthesized via ball milling, and the E_{hull} is not significantly high, with no Li_2S detected. Additionally, the domain size is sufficiently large for the crystal structure to be identified using a lab-source XRD in Figure 1d. The anionic sizes of Cl and S are also similar (Cl^- = 1.81 Å and S^{2-} = 1.84 Å). If Li_2S were excessive, it would have been detected, but it was not. This result suggests that the phase is likely to have generally formed in the bulk near x = 0.8 (~18/23), making this assumption reasonable. In contrast to $0.8Li_2O–ZrCl_4$, because sulfur is incorporated into the bulk rather than the interface, the phase ratio between ZrO_2 and hcp in the oxygen-substituted system (~1:2) differs from that of ZrS_2 and ccp in the sulfur-substituted counterpart (~3:7), despite using the same amount of lithium source ($0.8Li_2A–ZrCl_4$). This ratio, confirmed by both computational predictions and experimental observations using PDF and EXAFS, further clarifies the difference in phase formation between oxygen- and sulfur-substituted systems.

Supplementary Table 20. The calculated values related to ionic conductivity (pre-factor σ_0, activation energy E_a, migration energy E_m, mobile carrier formation energy E_f, hopping frequency v, carrier concentration factor *C*, migration entropy ΔS_m) of hcp-Li_2ZrCl_6, 0.8Li_2O–$ZrCl_4$, ccp-Li_2ZrCl_6 and 0.8Li_2S–$ZrCl_4$.

	hcp-Li_2ZrCl_6	0.8Li_2O–$ZrCl_4$,	ccp-Li_2ZrCl_6	0.8Li_2S–$ZrCl_4$
σ (25 ºC)	3.7 × 10^{-4} S cm^{-1}	1.78 × 10^{-3} S cm^{-1}	4.3 × 10^{-6} S cm^{-1}	1.01 × 10^{-3} S cm^{-1}
σ_0	77848 S cm^{-1} K^{-1}	29836 S cm^{-1} K^{-1}	99451 S cm^{-1} K^{-1}	69324 S cm^{-1} K^{-1}
E_a	346.39 meV	280.96 meV	466.75 meV	317.22 meV
E_m	333.11 meV	276.03 meV	445.53 meV	315.04 meV
E_f	13.28 meV	4.93 meV	21.22 meV	2.18 meV
v	1.28 × 10^6 Hz at 0 °C	5.17 × 10^6 Hz at 0 °C	4.30 × 10^4 Hz at 0 °C	3.14 × 10^6 Hz at 0 °C
C	4.36 × 10^{-8} S cm^{-1} Hz^{-1} K	4.60 × 10^{-8} S cm^{-1} Hz^{-1} K	1.15 × 10^{-8} S cm^{-1} Hz^{-1} K	2.41 × 10^{-8} S cm^{-1} Hz^{-1} K
ΔS_m	1.56 × 10^{-4} eV K^{-1}	2.46 × 10^{-4} eV K^{-1}	4.42 × 10^{-5} eV K^{-1}	1.38 × 10^{-4} eV K^{-1}

Supplementary Text 8. Ionic conduction properties

The enhanced ionic conductivity in 0.8Li_2O–$ZrCl_4$ primarily originates from a significant reduction in migration energy rather than a substantial increase in mobile carrier concentration. Although the formation of mobile carriers becomes more favorable due to the lower formation energy, the overall carrier concentration remains comparable to hcp-LZC, likely due to partial immobilization of Li^+ near strongly coordinating O^{2-} sites. Instead, the key contributing factors are the increased hopping frequency and migration entropy, reflecting enhanced lattice distortion and dynamic disorder. This indicates a substantial distortion of the lattice and hopping site. In sulfur incorporation, the enhanced ionic conductivity in 0.8Li_2S–$ZrCl_4$ stems from both a markedly reduced migration energy and a drastically lower mobile carrier formation energy compared to ccp-LZC. Unlike the oxygen-substituted system, sulfur substitution not only facilitates easier carrier formation but also significantly increases the number of mobile Li^+ ions participating in conduction. This is further supported by the substantial rise in hopping frequency, carrier concentration factor, and migration entropy, all of which indicate a pronounced enhancement in ion dynamics. These improvements collectively reflect a fundamental transformation of the ccp lattice, wherein sulfur substitution induces interlayer expansion and dynamic lattice flexibility, effectively activating a framework that is otherwise unfavorable for Li^+ migration.

Supplementary Table 21. Lattice parameters and lattice volume of hcp-$Li_{2+x}ZrCl_{6-x}O_x$ (x = 0, 0.166, 0.333, 0.5, 0.667, 0.833, and 1).

Composition	a (Å)	b (Å)	c (Å)	α (°)	β (°)	γ (°)	Volume (Å^3)
(Li_2ZrCl_6) $Li_{12}Zr_6Cl_{36}$	12.1126	11.0744	11.0744	60	90	90	1286.495
(x=0.166) $Li_{13}Zr_6Cl_{35}O_1$	12.2484	10.9911	11.1114	59.8875	89.5377	88.7671	1293.666
(x=0.333) $Li_{14}Zr_6Cl_{34}O_2$	12.3846	11.2154	10.9683	59.2517	87.7419	89.9996	1307.930
(x=0.5) $Li_{15}Zr_6Cl_{33}O_3$	12.4547	10.9096	11.1836	59.3349	89.9016	88.9252	1306.794
(x=0.667) $Li_{16}Zr_6Cl_{32}O_4$	12.4427	10.8767	11.2142	60.9901	88.8953	89.9994	1326.949
(x=0.833) $Li_{17}Zr_6Cl_{31}O_5$	12.3473	10.7651	11.2471	60.4349	89.4990	90.3996	1300.147
(x=1) $Li_{18}Zr_6Cl_{30}O_6$	12.2735	10.6472	11.1284	61.4198	90.1507	89.9999	1277.033

Supplementary Table 22. Lattice parameters and lattice volume of ccp-$Li_{2+x}ZrCl_{6-x}S_x$ (x = 0, 0.125, 0.25, 0.375, 0.5, 0.625, 0.75, 0.875).

Composition	a (Å)	b (Å)	c (Å)	α (°)	β (°)	γ (°)	Volume ($Å^3$)
(Li_2ZrCl_6) $Li_{16}Zr_8Cl_{48}$	13.0702	11.0627	13.3395	90	111.1977	90	1798.276
(x=0.125) $Li_{17}Zr_8Cl_{47}S_1$	12.8853	11.1238	13.5316	90.2548	108.4886	90.0348	1839.399
(x=0.25) $Li_{18}Zr_8Cl_{46}S_2$	12.8854	11.1126	13.4645	90.9988	108.5971	90.1027	1826.975
(x=0.375) $Li_{19}Zr_8Cl_{45}S_3$	12.8784	11.1332	13.3638	89.5763	109.2645	89.9385	1808.725
(x=0.5) $Li_{20}Zr_8Cl_{44}S_4$	12.8846	11.1467	13.3277	90.5613	108.8994	90.0514	1810.841
(x=0.625) $Li_{21}Zr_8Cl_{43}S_5$	12.8982	11.1363	13.2927	90.7243	109.1034	90.1092	1803.966
(x=0.75) $Li_{22}Zr_8$ $Cl_{42}S_6$	12.8907	11.144	13.2613	90	108.4688	90	1806.966
(x=0.875) $Li_{23}Zr_8Cl_{41}S_7$	12.8897	11.1197	13.1077	89.8789	108.4863	89.8702	1781.762

Supplementary Table 23. Continuous Symmetry Measure (CSM) values of Li octahedral site in hcp-Li_2ZrCl_6 with $Li_{2.5}ZrCl_{5.5}O_{0.5}$ and ccp-Li_2ZrCl_6 with $Li_{2.25}ZrCl_{5.75}S_{0.25}$. CSM = 0 corresponds to a perfectly symmetric coordinated environment and CSM= 66.7 corresponds to infinite elongation along one direction.

Index	hcp-Li_2ZrCl_6	hcp-$Li_{2.5}ZrCl_{5.5}O_{0.5}$	ccp-Li_2ZrCl_6	ccp-$Li_{2.25}ZrCl_{5.75}S_{0.25}$
Li1	0.242	0.411	0.291	0.345
Li2	0.242	1.627	0.301	0.360
Li3	0.242	0.587	0.301	0.292
Li4	0.242	1.374	0.283	0.312
Li5	0.242	23.268	0.306	0.410
Li6	0.242	5.965	0.291	0.345
Li7	0.242	2.361	0.306	0.321
Li8	0.242	0.749	0.308	0.517
Li9	0.242	3.596	0.306	0.749
Li10	0.242	1.713	0.308	0.749
Li11	0.242	5.241	0.306	0.410
Li12	0.242	0.707	0.301	0.517
Li13		2.660	0.292	0.321
Li14		0.527	0.283	0.360
Li15		0.679	0.301	0.278
Li16			0.292	0.312
Li17				0.292
Li18				0.278
Averaged CSM value	0.242	3.431	0.299	0.398

Supplementary Table 24. Lattice parameters and lattice volume of crystal structure of 1SD-hcp (Li_2ZrCl_6 and $Li_{2.5}ZrCl_{5.5}O_{0.5}$), FD-hcp ($Li_2ZrCl_6$ and $Li_{2.5}ZrCl_{5.5}O_{0.5}$), 1SD-ccp ($Li_2ZrCl_6$ and $Li_{2.25}ZrCl_{5.75}S_{0.25}$) and FD-ccp ($Li_2ZrCl_6$ and $Li_{2.25}ZrCl_{5.75}S_{0.25}$). 1 side-disorder (1SD) means cation disorder occurs asymmetrically near one side of the M2/M3 sites, and M3-site fully disordered (FD) indicates that the M3 sites are entirely filled with metal cations.

Structure	Composition	a (Å)	b (Å)	c (Å)	α (°)	β (°)	γ (°)	Volume (Å^3)
1SD	$Li_{12}Zr_6Cl_{36}$	11.035	11.035	11.792	90	90	60	1243.554
hcp	$Li_{15}Zr_6Cl_{33}O_3$	10.988	10.888	12.210	90.282	89.957	60.831	1275.517
FD	$Li_{12}Zr_6Cl_{36}$	11.019	11.019	11.576	90	90	60	1217.247
hcp	$Li_{15}Zr_6Cl_{33}O_3$	11.082	10.849	11.8120	89.438	90.887	61.258	1245.564
1SD	$Li_{16}Zr_8Cl_{48}$	12.795	11.083	12.338	90.093	91.672	89.993	1748.906
ccp	$Li_{18}Zr_8S_2Cl_{46}$	12.793	11.095	12.387	89.642	90.80928	89.814	1758.048
FD	$Li_{16}Zr_8Cl_{48}$	12.744	11.055	11.995	90.014	90.80654	89.994	1689.794
ccp	$Li_{18}Zr_8S_2Cl_{46}$	12.783	11.011	12.093	90.003	90.97785	90.013	1701.934

Supplementary Table 25. Key features derived from both experimental and computational analyses in $0.8Li_2O–ZrCl_4$ and $0.8Li_2S–ZrCl_4$. SRO means short-range ordering.

	$0.8Li_2O–ZrCl_4$	$0.8Li_2S–ZrCl_4$
Analysis		
Lab XRD	Weak peak	ccp or LiCl
Synchrotron XRD	hcp trigonal, residual Li_2O	ccp monoclinic
PDF	hcp trigonal, SRO of ZrO_2	hcp trigonal, SRO of ZrS_2
XANES	hcp-like Zr environment	ccp-like Zr environment
EXAFS	Zr-Cl bond elongation by O	Zr-Cl bond elongation by S
Raman	ZrX_6 polyhedral feature	ZrX_6 polyhedral feature
HRTEM	ZrO_2, hcp trigonal	ccp monoclinic
Phase identification		
Domain size	Nano-crystalline	Micro-crystalline
Observed residual component	Li_2O, ZrO_2	ZrS_2 (PDF)
Divalent-anion substitution	Oxygen gradient based interfacial substitution of hcp trigonal	Bulk substitution with phase transition from hcp trigonal to ccp monoclinic
Conduction Property		
σ (25 ℃)	1.78×10^{-3} S cm^{-1}	1.01×10^{-3} S cm^{-1}
E_a	280.96 meV	317.22 meV
σ_0	29836 S cm^{-1} K^{-1}	69324 S cm^{-1} K^{-1}
E_m	276.03 meV	315.04 meV
E_f	4.93 meV	2.18 meV
v	5.17×10^{6} Hz at 0 °C	3.14×10^{6} Hz at 0 °C
C	4.60×10^{-8} S cm^{-1} Hz^{-1} K	2.41×10^{-8} S cm^{-1} Hz^{-1} K
$\Delta \boldsymbol{S_m}$	2.46×10^{-4} eV/K	1.38×10^{-4} eV/K
DFT Calculations		
Diffusivity	3.56×10^{-6} cm^2 s^{-1}	5.49×10^{-7} cm^2 s^{-1}
Structural feature	Divalent anion clustering	Divalent anion clustering
Probability density	Enhanced 3D connectivity	Enhanced ab plane conduction
Lattice volume increase	1.58 %	1.60 %
Avg. polyhedral distortion index	0.047 (More distorted)	0.009
$ICOHP_{Avg}$ value	-0.3700 (More weekends, more diversified)	-0.3749
CSM_{Avg} value	3.431 (More distorted)	0.398
Dominant mechanism	Severe Li site distortion	Activation of ab plane conduction
Common Feature	Divalent anion clustering, charge carrier increase, lattice expansion, lattice distortion, Li site distortion, Li-Cl bond weakening, site energy diversification, energy landscape flattening	

Supplementary Table 26. DC measurement result and parameters for electronic conductivity of $0.8Li_2O–ZrCl_4$, $0.8Li_2S–ZrCl_4$ and hcp-Li_2ZrCl_6.

Sample	D (cm)	A (cm^2)	I (A)	V (V)	R (Ω)	t (cm)	σ_{e-} ($S\ cm^{-1}$)	$\sigma_{e-\ (Avg)}$ ($S\ cm^{-1}$)
$0.8Li_2O$-$ZrCl_4$	1.3	1.327	2.07×10^{-9}	0.1	4.83×10^{7}	0.037	5.77×10^{-10}	4.60×10^{-10}
			3.22×10^{-9}	0.2	6.21×10^{7}		4.49×10^{-10}	
			3.81×10^{-9}	0.3	7.87×10^{7}		3.54×10^{-10}	
$0.8Li_2S$-$ZrCl_4$			1.08×10^{-9}	0.1	9.26×10^{7}	0.045	3.66×10^{-10}	3.32×10^{-10}
			2.03×10^{-9}	0.2	9.85×10^{7}		3.44×10^{-10}	
			2.53×10^{-9}	0.3	1.19×10^{8}		2.86×10^{-10}	
hcp-Li_2ZrCl_6			7.22×10^{-9}	0.1	1.39×10^{8}	0.048	2.61×10^{-10}	2.72×10^{-10}
			1.60×10^{-9}	0.2	1.25×10^{8}		2.89×10^{-10}	
			2.21×10^{-9}	0.3	1.36×10^{8}		2.66×10^{-10}	
