# Supplementary material for: Divalent anion-driven framework regulation in Zr-based halide solid electrolytes for all-solid-state batteries
Source: Nat Commun. 2025 Nov 27;16:10678. doi: 10.1038/s41467-025-65702-2 (PMC12660725; doi:10.1038/s41467-025-65702-2)
Supplement: Supplementary file 3 — Description of Additional Supplementary Files [file 41467_2025_65702_MOESM3_ESM.pdf]

Description of Additional Supplementary Files

Supplementary Data 1. Optimized crystal structure of hcp-Li_2ZrCl_6.

Supplementary Data 2. Optimized crystal structure of $Li_{2.167}ZrCl_{5.833}O_{0.167}$.

Supplementary Data 3. Optimized crystal structure of $Li_{2.333}ZrCl_{5.667}O_{0.333}$.

Supplementary Data 4. Optimized crystal structure of $Li_{2.5}ZrCl_{5.5}O_{0.5}$.

Supplementary Data 5. Optimized crystal structure of $Li_{2.5}ZrCl_{5.5}O_{0.5}$.

Supplementary Data 6. Optimized crystal structure of $Li_{2.667}ZrCl_{5.333}O_{0.667}$.

Supplementary Data 7. Optimized crystal structure of $Li_{2.833}ZrCl_{5.167}O_{0.833}$.

Supplementary Data 8. Optimized crystal structure of ccp-Li_2ZrCl_6.

Supplementary Data 9. Optimized crystal structure of $Li_{2.125}ZrCl_{5.875}S_{0.125}$.

Supplementary Data 10. Optimized crystal structure of $Li_{2.25}ZrCl_{5.75}S_{0.25}$.

Supplementary Data 11. Optimized crystal structure of $Li_{2.375}ZrCl_{5.625}S_{0.375}$.

Supplementary Data 12. Optimized crystal structure of $Li_{2.5}ZrCl_{5.5}S_{0.5}$.

Supplementary Data 13. Optimized crystal structure of $Li_{2.625}ZrCl_{5.375}S_{0.625}$.

Supplementary Data 14. Optimized crystal structure of $Li_{2.75}ZrCl_{5.25}S_{0.75}$.

Supplementary Data 15. Optimized crystal structure of $Li_{2.875}ZrCl_{5.125}S_{0.875}$.

Supplementary Data 16. Initial atomic configuration of hcp-Li_2ZrCl_6 from AIMD simulation at 600 K.

Supplementary Data 17. Final atomic configuration of hcp-Li_2ZrCl_6 from AIMD simulation at 600 K after 300ps.

Supplementary Data 18. Initial atomic configuration of $Li_{2.5}ZrCl_{5.5}O_{0.5}$ from AIMD simulation at 600 K.

Supplementary Data 19. Final atomic configuration of $Li_{2.5}ZrCl_{5.5}O_{0.5}$ from AIMD simulation at 600 K after 300ps.

Supplementary Data 20. Initial atomic configuration of ccp-Li_2ZrCl_6 from AIMD simulation at 600 K.

Supplementary Data 21. Final atomic configuration of ccp-Li_2ZrCl_6 from AIMD simulation at 600 K after 300ps.

Supplementary Data 22. Initial atomic configuration of $Li_{2.25}ZrCl_{5.75}S_{0.25}$ from AIMD simulation at 600 K.

Supplementary Data 23. Final atomic configuration of $Li_{2.25}ZrCl_{5.75}S_{0.25}$ from AIMD simulation at 600 K after 300ps.
